# Transcriptome Profiling Provides Insights Into Potential Antagonistic Mechanisms Involved in *Chaetomium globosum* Against *Bipolaris sorokiniana*

**DOI:** 10.3389/fmicb.2020.578115

**Published:** 2020-12-07

**Authors:** K. Darshan, Rashmi Aggarwal, Bishnu Maya Bashyal, Jagmohan Singh, V. Shanmugam, Malkhan S. Gurjar, Amolkumar U. Solanke

**Affiliations:** ^1^Fungal Molecular Biology Laboratory, Division of Plant Pathology, ICAR—Indian Agricultural Research Institute, New Delhi, India; ^2^ICAR-National Institute for Plant Biotechnology, ICAR-IARI, New Delhi, India

**Keywords:** *Bipolaris sorokiniana*, biocontrol, *Chaetomium globosum*, Illumina HiSeq, RNA-seq, transcriptome

## Abstract

*Chaetomium globosum* Kunze is recognized as a potential biocontrol fungus against spot blotch of wheat caused by *Bipolaris sorokiniana*. Its molecular mechanism of biocontrol activity and the biosynthetic pathways involved have not been yet elucidated. Here, global transcriptome profiling of *C. globosum* strain Cg2 during interaction with *B. sorokiniana* isolate BS112 using RNA-seq was performed in order to gain insights into the potential mechanisms of antagonism. The Illumina HiSeq platform (2 × 150 bp) yielded an average of 20–22 million reads with 50–58% GC. *De novo* assembly generated 45,582 transcripts with 27,957 unigenes. Transcriptome analysis displayed distinct expression profiles in the interaction (Cg2–BS112), out of which 6,109 unique differentially expressed genes were present. The predominant transcripts classified as genes involved in “catalytic activity” constituted 45.06%, of which 10.02% were associated with “hydrolytic activity” (GO:0008152), and similarly, in the biological process, 29.18% of transcripts were involved in “metabolic activity” (GO:0004096 and GO:0006979). Heat map and cluster categorization suggested an increase in the expression levels of genes encoding secondary metabolites like polyketide synthase (GO:0009058), *S*-hydroxymethyl glutathione dehydrogenase (GO:0006069), terpene cyclase (EC 4.2.3.-), aminotran_1_2 domain-containing protein (GO:0009058), and other hydrolytic CAZYmes such as the glycosyl hydrolase (GH) family (GH 13, GH 2, GH 31, and GH 81; GO:0005975), cellulase domain-containing protein, chitinases, β-1, 3-glucanases (GO:0004565), glucan endo-1,3-beta-glucanase (GO:0052861), and proteases (GO:0004177). The obtained RNA-seq data were validated by RT-qPCR using 20 randomly chosen genes, showing consistency with the RNA-seq results. The present work is worldwide the first effort to unravel the biocontrol mechanism of *C. globosum* against *B. sorokiniana*. It generated a novel dataset for further studies and facilitated improvement of the gene annotation models in the *C. globosum* draft genome.

## Introduction

Spot blotch of wheat caused by *Bipolaris sorokiniana* (Sacc.) Shoem. (teleomorph, *Cochliobolus sativus*) has become a major destructive fungal disease of wheat (*Triticum aestivum* L.). The pathogen is seedborne and soilborne, making it one of the most damaging pathogens in the warm and humid wheat-growing belts of the world (Gupta et al., 2018). Geographically, its infection ranges from the Eastern Gangetic plains of India to Southeast Asia, China, Africa, and Latin America (Joshi et al., 2007; Acharya et al., 2011; Gupta et al., 2018). Upon infection, the pathogen produces foliar lesions, grain discoloration, seedling blight, and root rot (Duveiller et al., 2005). Among the different types of biotic symptoms induced, the foliar spot blotch is considered as a major constraint, which hugely hampers wheat and barley production on a global scale. Globally, the spot blotching disease affects about 25 million hectares of wheat-cultivated land (Van Ginkel and Rajaram, 1998). In South Asia, the annual yield loss of wheat due to this disease is up to 15–25% (Dubin and van Ginkel, 1991; Duveiller and Sharma, 2009). More importantly, in India, most farmers' lands are devoted to the rice–wheat cropping system (Nagarajan and Kumar, 1998), which ultimately reduces grain yield even up to 25% in severe cases (Saari, 1998; Duveiller and Sharma, 2009). Unfortunately, with changes in the cropping system, intensity, management practices, and climate, the foliar blight complex has now been established as a serious disease in both northeastern and western India (Sharma et al., 2007; Aggarwal et al., 2010). Therefore, controlling this disease in a country assumes greater importance where at least 200 million Indians go to bed hungry every night (Assocham, 2015).

For the last 25 years, chemical fungicides have been used as a preventive and therapeutic measure to manage this disease, but, due to the ill effects of chemicals on human health and the environment and the evolution of multiresistant strains in pathogens, use of synthetic fungicides is increasingly being restricted (Hollomon, 2015a,b). Consequently, researchers have focused their efforts on developing alternative methods. One of the alternative approaches to address these risks is biological methods that are biodegradable and environmentally friendly in nature and that have received tremendous attention worldwide in recent years (Maksimov et al., 2011). In this direction, the biocontrol fungus *Chaetomium globosum* (Phylum Ascomycota), an endophyte, has gotten international recognition due to its adaptability to extreme environments. This fungus is a filamentous, saprophytic, and well-known mesophilic member of the family Chaetomiaceae established by Kunze in 1817 (Von et al., 1986). It produces lemon-shaped olive brown ascospores in globose to pyriform-shaped perithecia clothed with irregularly coiled appendages (Domsch et al., 1993). Multiple reports illustrate the potential of *C. globosum* as a biocontrol agent. It mycoparasitizes and produces a variety of antifungal metabolites such as chaetoglobosins A and C, chaetomin, cochliodinol, chaetosin, chaetomugilins D and A, and prenisatin (Qin et al., 2009; Aggarwal et al., 2013; Zhang et al., 2013), which suppress the growth of many soilborne and seedborne phytopathogens including *B. sorokiniana* (Aggarwal et al., 2004; Istifadah and McGee, 2006). Furthermore, antagonistic mechanisms of this fungus are exerted through competition for space and nutrients (Vannacci and Harman, 1987) as well as mycoparasitism (Mandal et al., 1999). At the microscopic level, the host pathogen mycelium shows deformed conidia with distorted walls, lysis, and formation of holes, inhibiting conidial germination and hyphal elongation (Mandal et al., 1999; Biswas et al., 2000; Moya et al., 2016). Additionally, it also contributes to plant health by acting as a plant growth-promoting fungus (PGPF) and by inducing systemic resistance in wheat against *B. sorokiniana* and *Puccinia graminis* f. sp. *tritici* (Aggarwal, 2015). It is also reported to produce phosphatases and phytases, mobilize P, and enhance castor, wheat, and pearl millet production (Vaghasia et al., 2017). More recently, the fungus has been introduced into the world of nanobiotechnology by Singh et al. (2018) for synthesizing and characterizing antimicrobial silver and gold nanoparticles using aq. cell-free filtrate (CFF) of *C. globosum*.

Antagonistic activity of *C. globosum* was reported against various fungal plant pathogens (Biswas et al., 2000; Soytong et al., 2001; Aggarwal et al., 2004; Aggarwal, 2015). Among them, our research group has attempted a proteomics approach to identify differentially expressed proteins from *C. globosum* (Sharma et al., 2014). Recently, the draft genome sequence of a *C. globosum* isolate CBS 148.51 (ATCC 6205) was sequenced and released by Genome Announcements (Cuomo et al., 2015). However, the molecular mechanisms of antagonism, patterns of gene expression at the molecular level, and identification of biosynthetic pathways are not yet explored in *C. globosum*. Transcriptome approaches have been occasionally applied to the study of biological control mechanisms (Gkarmiri et al., 2015; Hennessy et al., 2017; Liu et al., 2019). The present work is the first effort worldwide to unravel biocontrol mechanisms to report their probable role in the antagonism of potential *C. globosum* strain Cg2 against the phytopathogenic fungus *B. sorokiniana* isolate BS112 using RNA-seq approach. This study reveals the inhibitory mechanisms of *C. globosum* against *B. sorokiniana* plant pathogen in general. This will enable us to genetically improve the most significant *C. globosum* strain to be formulated as a biopesticide.

## Materials and Methods

### Fungal Strains and Culture Conditions

The potential biocontrol strain of *C. globosum* Cg2 (ITS NCBI accession AY429049) isolated earlier from wheat leaf surface (Mandal et al., 1999; Aggarwal et al., 2004) and a *B. sorokiniana* aggressive isolate BS112 isolated from northeastern plain zone (ITS NCBI accession KU201275) were maintained in the Fungal and Molecular Biology Laboratory at Division of Plant Pathology, Indian Agricultural Research Institute (IARI), New Delhi. Cultures of the Cg2 and BS112 were also deposited in the Indian Type Culture Collection (ITCC), New Delhi, with ITCC accession nos. 6210 and 8841, respectively. The strains were revived on potato dextrose agar (PDA) medium at 25 ± 2°C by single spore isolation. The cultures were maintained on PDA slants and used in various experiments.

### *In vitro* Dual-Culture Assay

The confrontation assay between *C. globosum* (Cg2) and *B. sorokiniana* (BS112) strains was carried out in dual-culture assay as previously described (Rubio et al., 2009) with some modifications. Nunc™ OmniTray™ sterile plates (Fisher Scientific, Denmark) (9-cm diameter) containing 20 ml sterilized PDA medium were uniformly covered with a sterilized cellophane membrane (Biotech Desk, Hyderabad, India). Initially, the mycelial agar disk (8-mm diameter) was taken from the 7-day-old cultures of *C. globosum* using a cork borer and placed at one side of the petri dish (1 cm from the edge of the plate). Subsequently, 7-day-old mycelial disk (8-mm diameter) of *B. sorokiniana* was placed on the opposite side of the same petri dish (1 cm away from the margin of the opposite side of the plate). The petri plates contained only the pathogen, and biocontrol disks were separately maintained as control. The inoculated plates were kept it for incubation at 25 ± 2°C under continuous fluorescent light. The *C. globosum* mycelia from *C. globosum* control and *B. sorokiniana*–*C. globosum* interaction plates were harvested in aluminum foil after 3, 5, 7, 9, and 11 days of inoculation and immediately frozen in liquid N_2_ and stored at −80°C until RNA isolation. This experiment was performed in two replicates for each growth period, with mycelia subsequently pooled to form composite samples.

### Microscopic Image Analysis of Dual-Culture Plates

SEM analysis of dual-culture plates of *C. globosum* with *B. sorokiniana* was conducted following the procedure of Pisi et al. (2001). Small pieces of agar were taken from dual cultures at the inhibition zones of *B. sorokiniana*. Fixation of specimens was done by using chilled (4°C) 2.5% glutaraldehyde (Sigma-Aldrich) prepared in 0.1 M phosphate buffer for 2 h at room temperature followed by three washes each for 15 min, in 0.1 M phosphate buffer (Fisher Scientific) at a pH of 7.4. After 12 h of refrigeration, fixed samples were dehydrated in a graded ethanol series (30, 50, 70, 80, 95, and 100% ethanol, 15 min each). Samples were chemically dried using hexamethyldisilazane (Sigma-Aldrich). The critically dried samples were then adhered on to the aluminum specimen mounts with colloidal silver paste and then sputter-coated with 24-nm gold palladium. Samples were examined and photographed on a scanning electron microscope (SEM) (Zeiss, EVO MA10) at an accelerating voltage of 20 kV/EHT and 10 Pa.

For light microscopic studies, fungal colonies were picked up by the inoculating needle and placed on the slide mounted in 50% (v/v) glycerol and then covered with a coverslip. The slides were observed under a light microscope (Olympus, CH2Oi).

### RNA Extraction, cDNA Library Preparation, and Next-Generation RNA Sequencing

The total RNA was extracted from frozen mycelial samples using Qiagen RNeasy Plant Mini Kit (QIAGEN, Germany) in accordance with the manufacturer's instructions. The concentration and purity of the RNA samples were checked and quantified using a spectrophotometer (NanoDrop ND-1000; Thermo Fisher Scientific, Waltham, MA, USA). The RNA quality of all samples was tested on 1% agarose gel. The extracted RNA was assessed for its quality and quantity using RNA Pico chip on the Agilent Bioanalyzer 2100 system (Agilent Technologies, CA, USA) (Supplementary Table 1). Four micrograms of total RNA per sample was used to construct a transcriptome library using NEBNext® Ultra RNA Library Prep Kit for Illumina® (NEB, Ipswich, MA, USA) according to the manufacturer's protocol using a TruSeq adaptor to index each sample during the library preparation. The effective concentration of the library was then precisely quantified using a qPCR to ensure the library quality. The size of the purified library was measured on the Bioanalyzer 2100 using DNA 1000 Lab Chip. A library with an average size of more than 300 bp was taken for sequencing in an Illumina sequencing platform (HiSeq™ 2500) by Guangzhou Saizhe Biotechnology Co., Ltd. (Guangzhou, China) using Illumina HiSeq 151 × 2 paired-end (PE) read technology.

### Genome Guided *De novo* Transcriptome Assembly and Analysis

Raw reads with a Phred score of less than 20 were discarded using the Perl script prinseq-lite.pl v0.20.4 (https://sourceforge.net/projects/prinseq/) (-min_len ≤ 40 bp; -min qual_score ≤ 20) followed by the FastQC v0.11.7 visualization tool (https://www.bioinformatics.babraham.ac.uk/projects/fastqc/) to check the quality of the reads based on various parameters such as per-base sequence quality, per-sequence quality score, sequence length distribution, GC content, overrepresented sequences, biasness of the reads, and K-mer content (Miller et al., 2010). The contaminants such as the adapter sequence, reads with unknown nucleotides larger than 5%, the start and end of reads having a quality score ≤25 along with dropping off the reads below 50-bp length were removed using the Trimmomatic tool version 0.30 (Bolger et al., 2014) available at http://www.usadellab.org/cms/?page=trimmomatic to get clean reads. Genome-guided *de novo* assembly was done using Trinity assembler V2.4.0 (https://github.com/trinityrnaseq/trinityrnaseq/wiki) for all the Cg2 and BS112 samples based on algorithm *De Bruijn* graphs with default parameters: minimum contig length of 200, length fraction of 0.15, and similarity fraction of 0.95 with a bubble size of 50. The individual assembled sequences were clustered using CD-HIT (version 4.6) to remove redundancy at 90% sequence similarity. Unigenes were extracted using the inbuilt Trinity Perl script. Cg2 and BS112 clustered assemblies were combined and clustered again using CD-HIT for constructing a combined assembly of Cg2–BS112. Unigenes were extracted from this combined assembly. All samples were mapped using the BWA-MEM algorithm (version 0.7.5) against Cg2–BS112 unigenes with default parameters. The final assembled transcripts were used for further downstream analysis.

### Differential Analysis and Gene Annotation

The assembled reads were used to estimate gene expression, and the transcripts were quantified using the Cufflinks program module. The expression level of each of the genes was quantified by RNA-seq by expectation maximization (RSEM) tool (Li and Dewey, 2011) available at https://deweylab.biostat.wisc.edu/rsem/ in the form of fragments per kilobase of exon per million mapped reads (FPKM). The number of reads mapped to unigenes was calculated using SAMtools (version 0.1.19) for each sample. Differential analysis of all possible combinations was carried out by using DESeq 2 V 1.6.3 (https://support.bioconductor.org/packages/release/) with selected filters like *p*-values of 0.05 and log2FC. R package such as Cummerbund was performed to prepare heat maps (Goff et al., 2012), and hierarchical clustering was done using Euclidean correlation matrix. After DESeq analysis, ggplot2 was used to draw volcano plots (Wickham, 2016) with default parameters.

Functional annotations of individual and combined unigenes of samples were performed by aligning those unigenes to the non-redundant (NR) protein database (version 36) of NCBI employing BLASTX v2.2.31+ (Suzuki et al., 2015) using a threshold *E*-value of 1 × e^−3^. The assembled contigs were then functionally annotated by a Blast2GO software V 3.0 (https://www.blast2go.com) (Conesa et al., 2005). Further, the predicted proteins were subjected to pathway analysis using the Kyoto Encyclopedia of Genes and Genomes (KEGG) (Ogata et al., 1999) database to map the proteins involved in biochemical pathways. Gene ontology with a false discovery rate (FDR) value of approximately 0.05 has been used as a threshold to assess significant enrichment (Maere et al., 2005). The predicted proteins were categorized into three different functional groups, namely, biological process (BP), cellular component (CC), and molecular function (MF), with default parameters. Metabolic pathways related to significant genes were identified by using the KEGG database. The KEGG enrichment analysis of secondary metabolite-associated GO putative functions assigned by BLASTx to the unigenes was used identify the specific secondary metabolite biosynthesis unigenes. Significantly enriched biological components were used for network construction, and the biological relationship of the genes with associated pathways was deciphered by Cytoscape R packages (version 3.7.2) (Kohl et al., 2011). The biological functions of the specific genes of *C. globosum* were considered and investigated at a macro level.

### Validation of RNA-seq Data by qRT-PCR

To check the reliability of the RNA-seq data, 20 genes related to antagonism were selected based on FPKM values of transcripts and validated through quantitative RT-PCR (qRT-PCR) at three different time intervals. The fasta sequences of the selected differentially expressed genes (DEGs) were retrieved and fed into the online tool Primer3Plus (http://www.bioinformatics.nl/cgi-bin/primer3plus/primer3plus.cgi/) (You Frank et al., 2008) for designing the qRT-PCR primers. The selected primers were checked for the presence of secondary structures using an online-based tool IDT OligoanAlyzer (https://www.idtdna.com/analyzer/Applications/oligoanalyzer/). The primer sequences of selected genes used in the qRT-PCR analysis are listed in Supplementary Table 2. The selected primer sequences were synthesized by Eurofins Genomics (Bengaluru, India).

RNA samples were treated with DNase (QIAGEN DNase Max Kit, Germany) before cDNA synthesis. First-strand complementary DNA (cDNA) was synthesized by using Fermentas Thermo Scientific RevertAid first-strand cDNA synthesis kit. The reactions were assembled together into 48-well-plates. Each reaction (20-μl volume) contained 10 μl of SYBR Green Master Mix (Invitrogen, Carlsbad, CA), 1.0 μl of forward and reverse primers, 1 μl of diluted cDNA, and 7 μl of nuclease-free water. RT-qPCR was performed on a Bio-Rad MiniOpticon real-time PCR system (Applied Biosystems, Foster City, CA), and the thermal cycle reactions were as follows: 95°C for 10 min; 40 cycles of 95°C for 15 s, 58°C for 10 s, and 72°C for 15 s. All the tested samples were technically triplicated, and all the experiments were conducted with three biological replicates. The reference gene fungal glyceraldehyde 3-phosphate dehydrogenase (GAPDH) has been used for calibration in all experiments (Sharma et al., 2014). Expression ratios were calculated from the cycle threshold values using the 2^−ΔΔCT^ method (Kenneth and Thomas, 2001). The relative expressions of biocontrol-related genes in Cg2 control and Cg2–BS112 interaction were measured with respect to their control (NTC), and the endogenous control was used for normalization. The results obtained from RT-qPCR were compared with the results of RNA-seq-generated data.

## Results

### Inhibition of *B. sorokiniana* by *C. globosum*

**Figure 1:**
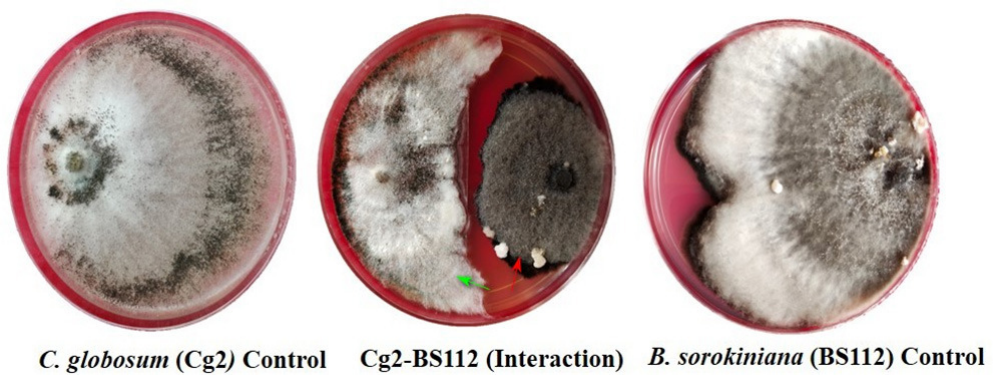
Dual-culture *in vitro* assay to study bipartite interactions between biocontrol fungus *C. globosum* (Cg2) (Green arrows) and *B. sorokiniana* (Red arrows).

**Figure 2:**
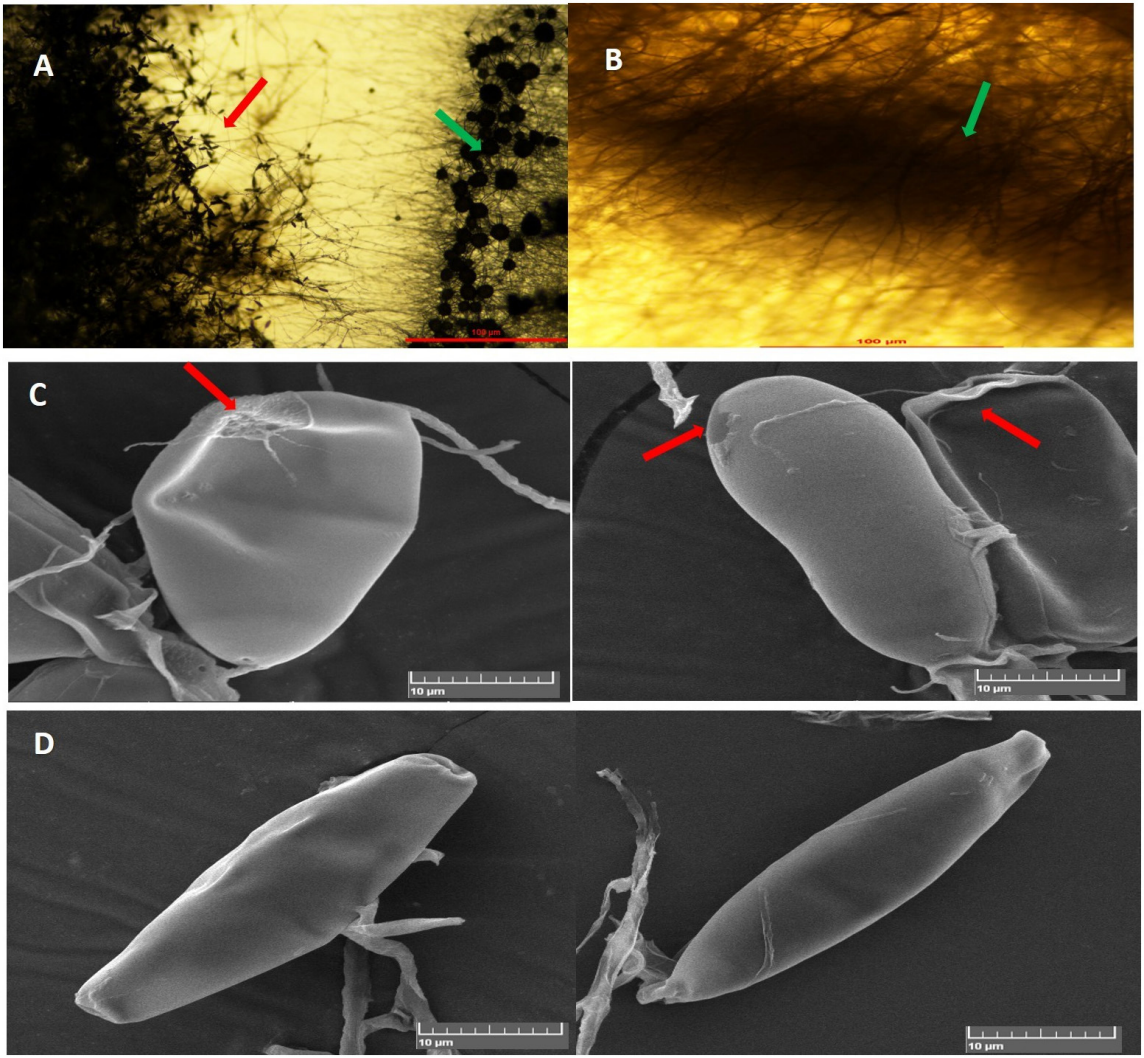
Antagonistic interactions of *C. globosum* (Cg2) against *B. sorokiniana* (BS112) in dual culture assay: **(A)** Inhibition zone due to antibiosis Red and green arrows indicating *B. sorokiniana* and *C. globosum*, respectively; **(B)** Orange pigmentation due to metabolite production. Bars **(A,B)** = 100 μM at 10×. **(C)** SEM images of plasmolysis and abnormal conidia of *B. sorokiniana* (Red arrows). **(D)** Conidia of *B. sorokiniana* (BS112) under control conditions, Bars **(C,D)** = 10 μM.

In dual-culture assays, *C. globosum* (Cg2) significantly inhibited the growth of the pathogen *B. sorokiniana* by producing a clear inhibition zone in relation to control. At 12 days after incubation, the percentage inhibition rate over control was 71.4% (Figure 1 and Supplementary Figure 1). Based on the morphological observations, the predominant mechanism of action of *C. globosum* against *B. sorokiniana* was antibiosis by producing a clear inhibition zone, and other mechanisms like mycoparasitism were also observed (Figure 2A). An orange pigmentation was observed in the interaction zone, which could be due to production of antifungal metabolites (Figure 2B). Based on scanning electron microscopic observation, *B. sorokiniana* displayed abnormal swellings and deformity in the conidia (Figure 2C) when compared with *B. sorokiniana* control (Figures 2C,D).

### Illumina Sequence Quality Analysis and Assembly

A total of six transcriptome libraries were sequenced to produce substantial sequencing data to identify genes linked to the antagonism. The Illumina HiSeq 2000 mRNA sequencing platform yielded an average of 19–23 million reads with an average read length of 343.66 bp. The GC content varies in the range of 50–58%. The transcriptome statistics of the six processed samples are given in Supplementary Table 3 and Supplementary Figure 2. The average number of clean reads per sample was 22,114,818 reads. Out of the total quality filtered reads, an average of 91.50% reads had a Phred score value ≥Q30, indicating that RNA-seq data are reliable and of good quality and can be used for further analysis.

All the processed reads that were analyzed from the three samples were assembled using Trinity (v2.4) software. A total of 55,173 transcripts were obtained, and further clustering of the assembled sequences using CD-HIT (version 4.6) to eliminate redundancy at 90% sequence similarity gives a total of 45,582 final transcripts with an average contig length of 2,322 bp, and an improved N50 value of 3,805 was obtained (Supplementary Table 4). The distribution of transcripts based upon length showed that around 64% (29,309) of transcripts were between 1,001 and 5,000 bp long followed by 11% (5022) of transcripts falling between 5,001 and 10,000 bp. The longest transcript length was 16,285 bp (Supplementary Table 4). All clean reads were mapped onto the reference genome of *C. globosum* available at the NCBI GenBank by following the reference genome-guided method. Aligning the reads to the reference genome showed that approximately 93–94% of the clean reads were aligned to the *C. globosum* genome, which indicates a better mapping percentage and can be used for gene expression analysis (Supplementary Figure 3). The RNA sequencing data were deposited at the SRA website/NCBI database/NCBI SRA database as BioSamples (accession numbers SRR11305503, SRR11305502, SRR113055501, SRR113055500, SRR11305499, and SRR11305498 under BioProject ID PRJNA612183).

### Characterization and Differential Expression Analysis Using the *In silico* Approach

In order to identify the genes that might be involved in antagonism during interaction with the pathogen, a total of 27,957 unigenes were blasted and functionally annotated using BLASTX. All the predicted unigenes of *C. globosum* were 100% annotated by comparing the obtained sequences with the available non-redundant protein sequences (Nr) database of NCBI, Gene Ontology (GO) terms, and Enzyme Commission (EC) numbers, showing 17,954 (64.20%), 12,016 (43.00%), and 1,526 (5.50%) transcripts, respectively. These predicted unigenes were significant hits primarily to their respective species, *C. globosum* (8,434) followed by a closely related cellulolytic fungus, *Myceliophthora thermophila* (3,825) (Supplementary Figure 4 and Supplementary Table 5).

**Figure 3:**
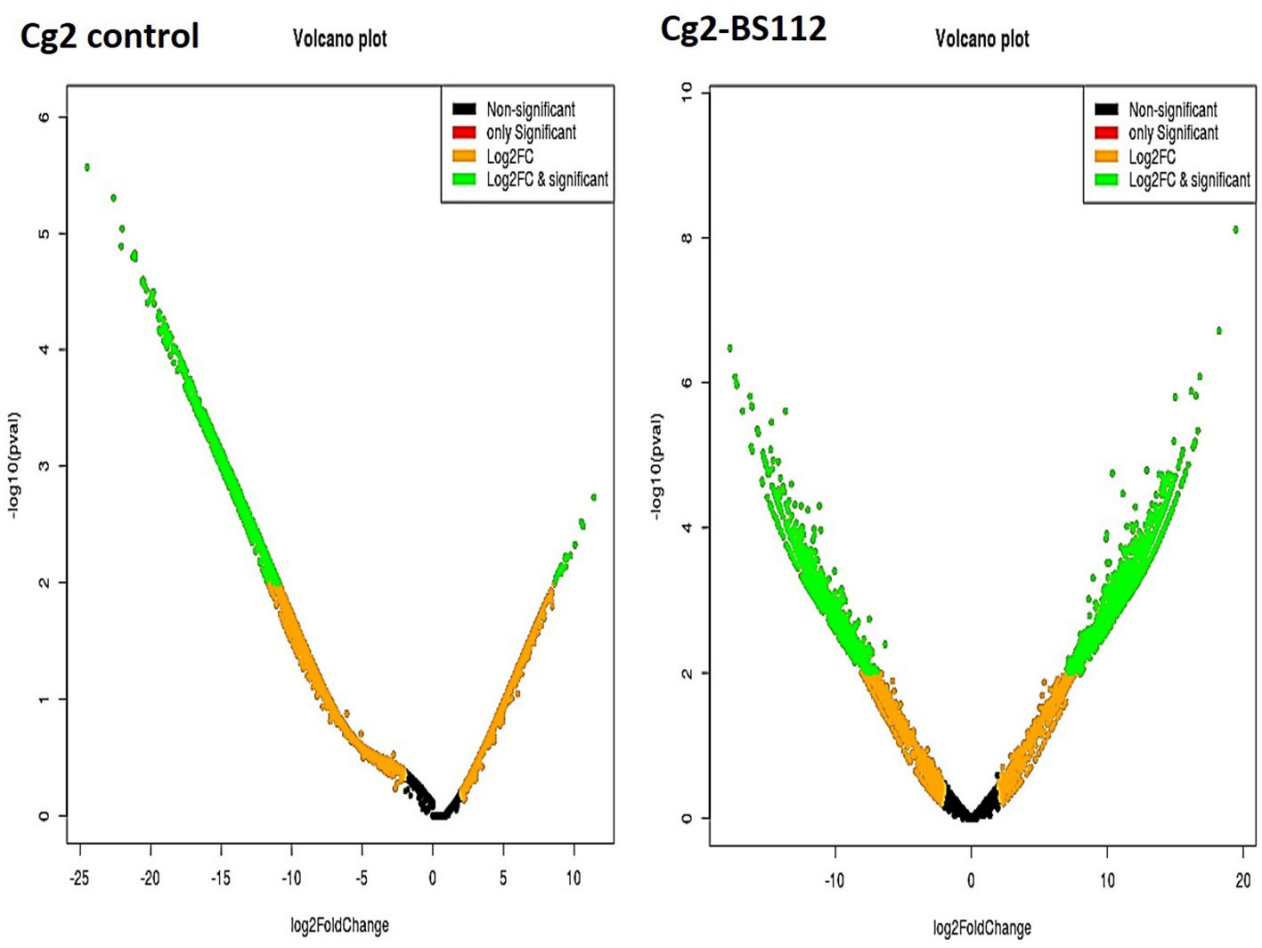
Volcano plot representing significant and non-significant DEGs based *P*-values. The green dot represents significantly differentially expressed genes.

The differential gene expression profile for *C. globosum* was performed using the most reliable DESeq v1.8.1 package. Based on FPKM values, which detected a total of 14,366 significant DEGs in *C. globosum* (Cg2) upon challenge with *B. sorokiniana* (BS112) (Supplementary Table 6). Volcano and scattered MA plot analysis of DEGs showed remarkable differences in gene distribution patterns, which were aptly delimited in the Cg2–BS112 interaction when compared with Cg2 control (Figure 3 and Supplementary Figure 5). Out of 14,366 DEGs, 7,145 and 7,221 transcripts were found to be significantly upregulated and downregulated genes, respectively (Supplementary Table 6). Among them, 6,109 were found to be unique DEGs for the Cg2–BS112 interaction, and 2,022 DEGs were common between Cg2–BS112 and Cg2 control as shown within the overlapping regions of the Venn diagram (Supplementary Figure 6). The commonly regulated genes are associated with structural development functions (integral component of the membrane [GO:0016021]; chitin synthase activity [GO:0004100]; and chitin biosynthetic process [GO:0006031]); and cellular development functions (cell [GO:0005623]; nucleus [GO:0005634]; and nucleolus [GO:0005730]) and enriched in the ribosome (large ribosomal subunit [GO:0015934] and translation [GO:0006412]) and mitochondria (mitochondrial inner membrane [GO:0005743; GO:0005739]), possibly participating in hyphal growth, cell wall integrity, and energy for sporogenesis, etc. Supplementary Table 7 represents the list of the top 20 commonly expressed genes along with the log2 fold change value and GO functions.

**Figure 4:**
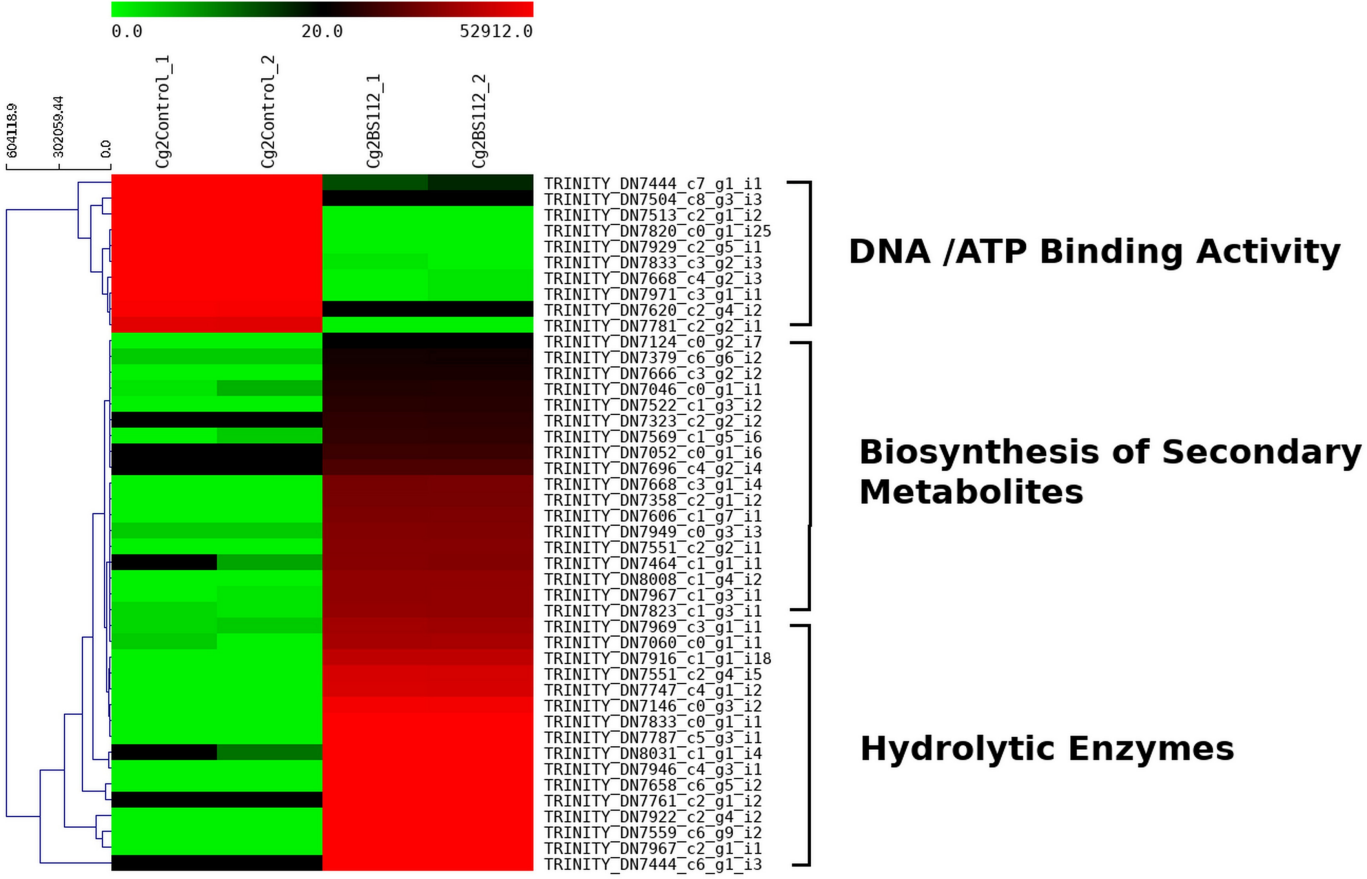
Heat map with cluster categorization representing the top 40 significant DEGs at three different comparisons of treatments. Each column represents the DEGs in different samples with two replicates. Red color shows upregulated and green color represents downregulated genes based on highest FPKM values. Each row represents an individual transcript.

Heat map and hierarchical cluster categorization of DEGs were generated to represent the global view of gene expression patterns and also depict their dynamic differences in the Cg2–BS112 interaction and Cg2 control condition (Figure 4). Higher percentages of genes related to hydrolytic enzymes and biosynthesis of secondary metabolites were observed in clusters 1 and 3 (Figure 4). This result suggests an increase in the expression levels of genes encoding secondary metabolites like polyketide synthase (biosynthetic process [GO:0009058]), non-ribosomal peptide (NRP) synthase (NRPS) (*S*-hydroxymethyl glutathione dehydrogenase [EC 1.1.1.284; GO:0006069]), terpene cyclase (EC 4.2.3.-), aminotran_1_2 domain-containing protein (biosynthetic process [GO:0009058]), 2-methoxy-6-polyprenyl-1,4-benzoquinol methylase, and other CAZYmes such as the GH family (GH 13, GH 2, GH 31, and GH 81) (hydrolase activity [GO:0005975]), cellulase domain-containing protein, chitinases, β-1,3 glucanases (beta-galactosidase activity [GO:0004565]), proteases (aminopeptidase activity [GO:0004177]), and metallopeptidase activity (GO:0008237), which were expressed in the Cg2–BS112 interaction (Table 1). Table 1 gives the list of the top 20 differentially expressed upregulated genes, which may support antagonism in *C. globosum* against the *B*. *sorokiniana* pathogen, based on the premise that *B. sorokiniana* mycelial inhibition occurs due to secretion of hydrolytic enzymes followed by release of antifungal secondary metabolites from *C. globosum*.

**Table 1:** List of differentially expressed upregulated genes in *C. globosum* (Cg2) upon challenge with *B. sorokiniana* (BS112).

**S. No**	**Gene ID**	**Name**	**Count value/ log fold change**	**Gene ontology**	**Function**	**Reference**
	XP_003664669.1	Protease	238,822.2	Serine-type endopeptidase activity [GO:0004252]	Protease, which can degrade the cell walls of fungi	Wu et al., 2017
	OAL48591.1	Fe2OG dioxygenase domain-containing protein	211,345.1	Iron ion binding [GO:0005506]; L-ascorbic acid binding [GO:0031418]; oxidoreductase activity, acting on paired donors, with incorporation or reduction of molecular oxygen [GO:0016705]	Catalytic role: important oxidizing biological catalysts (oxidoreductase activity), incorporation or reduction of molecular oxygen.	Farrow and Facchini, 2014
	D1ZHN6.2	Cyanate hydratase (Cyanase) (Cyanate lyase)	204,647.2	Cyanate hydratase activity [GO:0008824]; DNA binding [GO:0003677]; cyanate metabolic process [GO:0009439]	It involved in Nitrogen metabolism. The sole source of nitrogen nitrogen for growth	Walsh et al., 2000
	XP_003661882.1	Alpha-1,2-Mannosidase (EC 3.2.1.-)	192,714.9	Membrane [GO:0016020]; calcium ion binding [GO:0005509]; mannosyl-oligosaccharide 1,2-alpha-mannosidase activity [GO:0004571]; metabolic process [GO:0008152]	Signaling, maturation of N-glycans and cell wall degradation. (Mycoparasitism)	da Mota et al., 2016
	XP_003658861.1	Phosphoribosyla minoimidazole carboxylase (EC 4.1.1.21)	143,633.5	5-amino-4-imidazole carboxylate lyase activity [GO:0043727]; ATP binding [GO:0005524]; “*de novo*” IMP biosynthetic process [GO:0006189]	Metabolic pathways; Biosynthesis of secondary metabolites; Purine metabolism	Druzhinina and Kubicek, 2017
	XP_003649364.1	Carboxylic ester hydrolase (EC 3.1.1.-)	121,382.1	Hydrolase activity [GO:0016787]	Diverse group of hydrolases and lipase gene families. Lipid-degrading enzymes	Chen et al., 2016
	XP_003662561.1	ABC transporter-like protein	110,349.2	Integral component of membrane [GO:0016021]; plasma membrane [GO:0005886]; ATP binding [GO:0005524]; ATPase-coupled transmembrane transporter activity [GO:0042626]	Secretion of a wide variety of antagonism-related factors (toxins and enzymes) across biological membranes in response to pathogen	Ruocco et al., 2009
	XP_003664878.1	Polyketide synthase	28,276.0	Hydrolase activity, acting on ester bonds [GO:0016788]; phosphopantetheine binding [GO:0031177]; transferase activity [GO:0016740]; biosynthetic process [GO:0009058]	Required for production of antifungal polyketide. Involved in chaetoglobosin biosynthesis, pigmentation and sporulation	Yang et al., 2012
	XP_001222457.1	C2H2-type domain-containing protein	39,705.3	Integral component of membrane [GO:0016021]; nucleic acid binding [GO:0003676]	A fungal transcription factor essential for starch degradation and a regulator of glucose signaling and metabolism	Xiong et al., 2017
	OTA62198.1	Metalloprotease	50,977.5	Metallo-endopeptidase activity [GO:0004222]	Metallo-endopeptidase activity	Morán-Diez et al., 2019
	ABI95486.1	Endo-chitinase	37,909.5	Extracellular region [GO:0005576]; chitosanase activity [GO:0016977]; polysaccharide catabolic process [GO:0000272]	Defense against chitin-containing fungal pathogens, hydrolase activity, hydrolyzing O-glycosyl compounds	Yang et al., 2012
	XP_003664150.1	Glycosidase (EC 3.2.-.-)	66789.3	Fungal-type cell wall [GO:0009277]; incipient cellular bud site [GO:0000131]; hydrolase activity, [GO:0004553]; carbohydrate metabolic process [GO:0005975]; cell wall chitin metabolic process [GO:0006037]	Hydrolytic enzymes involved in mycoparasitism (cellobiose hydrolysis activities)	Tiwari et al., 2013
	AEQ54765.1	Catalase-peroxidase (CP) (EC 1.11.1.21)	28,747.02	Catalase activity [GO:0004096]; heme binding [GO:0020037]; metal ion binding [GO:0046872]; hydrogen peroxide catabolic process [GO:0042744]; response to oxidative stress [GO:0006979]	Peroxidases participate in diverse fungal secondary metabolism pathways and oxidizing various electron donors including NADP(H)	Zámocký et al., 2016
	KXX74964.1	Putative fungistatic metabolite	163,521.8	-	Inhibits the growth of the fungus *P. tritici-repentis*	Istifadah and McGee, 2006
	XP_003665898.1	P53-like transcription factor (Fragment)	45,726.4	DNA binding [GO:0003677]; DNA-binding transcription factor activity [GO:0003700]	Putative transcriptional activator that is a member of the Ndt80 family in the p53-like superfamily of proteins. Controls the production of extracellular proteases in response to starvation	Katz et al., 2013
	KXX77270.1	Cross-pathway control protein 1	303,336.2	DNA-binding transcription factor activity [GO:0003700]	Potential StzA binding sites were identified in cpcA (cross pathway control regulator of amino acid biosynthesis) and which makes link between intracellular amino acid availability and cellulase gene expression	Seiboth et al., 2012
	XP_001226572.1	Protein kinase domain-containing protein	34896.0/13.9537	ATP binding [GO:0005524]; protein serine/threonine kinase activity [GO:0004674]	Expression of cell-degrading enzyme	Wang et al., 2017
	KXX76280.1	Conidial yellow pigment biosynthesis polyketide synthase	8,232.6/11.18513	Hydrolase activity, acting on ester bonds [GO:0016788]; phosphopantetheine binding [GO:0031177]; biosynthetic process [GO:0009058]	Involved in melanin biosynthetic pathway in *C. globosum* and helps in conidia formation.	Hu et al., 2012
	XP_001223544.1	Glutamine synthetase	30,602.5/13.08425	Glutamate-ammonia ligase activity [GO:0004356]; glutamine biosynthetic process [GO:0006542]	Plays an essential role in the metabolism of nitrogen by catalyzing the condensation of glutamate and ammonia to form glutamine (Source of nitrogen as a nutrients)	Grabowska et al., 2012
	XP_001229654.1	Carboxypeptidase Y homolog A (EC 3.4.16.5)	34,363.4/14.66325	Vacuole [GO:0005773]; serine-type carboxypeptidase activity [GO:0004185]	Involved in degradation of small peptides.	Makino et al., 2019

### GO Functional and Pathway Enrichment Analysis of DEGs

GO functional enrichment analysis was performed to classify the predicted function of DEGs and their relationships between these transcripts. The analysis revealed that 11, 12, and 21 GO terms were grouped into the CC, MF, and BP, respectively (Supplementary Table 8). The predominant transcripts identified as genes involved in “catalytic activity” amounted to 45.06% followed by “nucleotide binding” (42.00%) activities of protein and DNA binding under MF. Out of the 45.06% of catalytic activity, 10.02% were involved in “hydrolytic activity” (GO:0008152), and similarly, in the BP, 29.18% of transcripts were involved in “metabolic activity” (GO:0004096 and GO:0006979) (Supplementary Figure 7). Reasonable transcripts were also involved in transportation, transcription, and biological regulation. About 18.1% of transcripts were active membranes and 17.24% in the membrane part. Other active CCs were cell parts (16.53%), active organelles (13.47%), organelle parts (7.24%), and protein-containing complexes (7.12%) (Supplementary Figure 7 and Supplementary Table 8).

**Figure 5:**
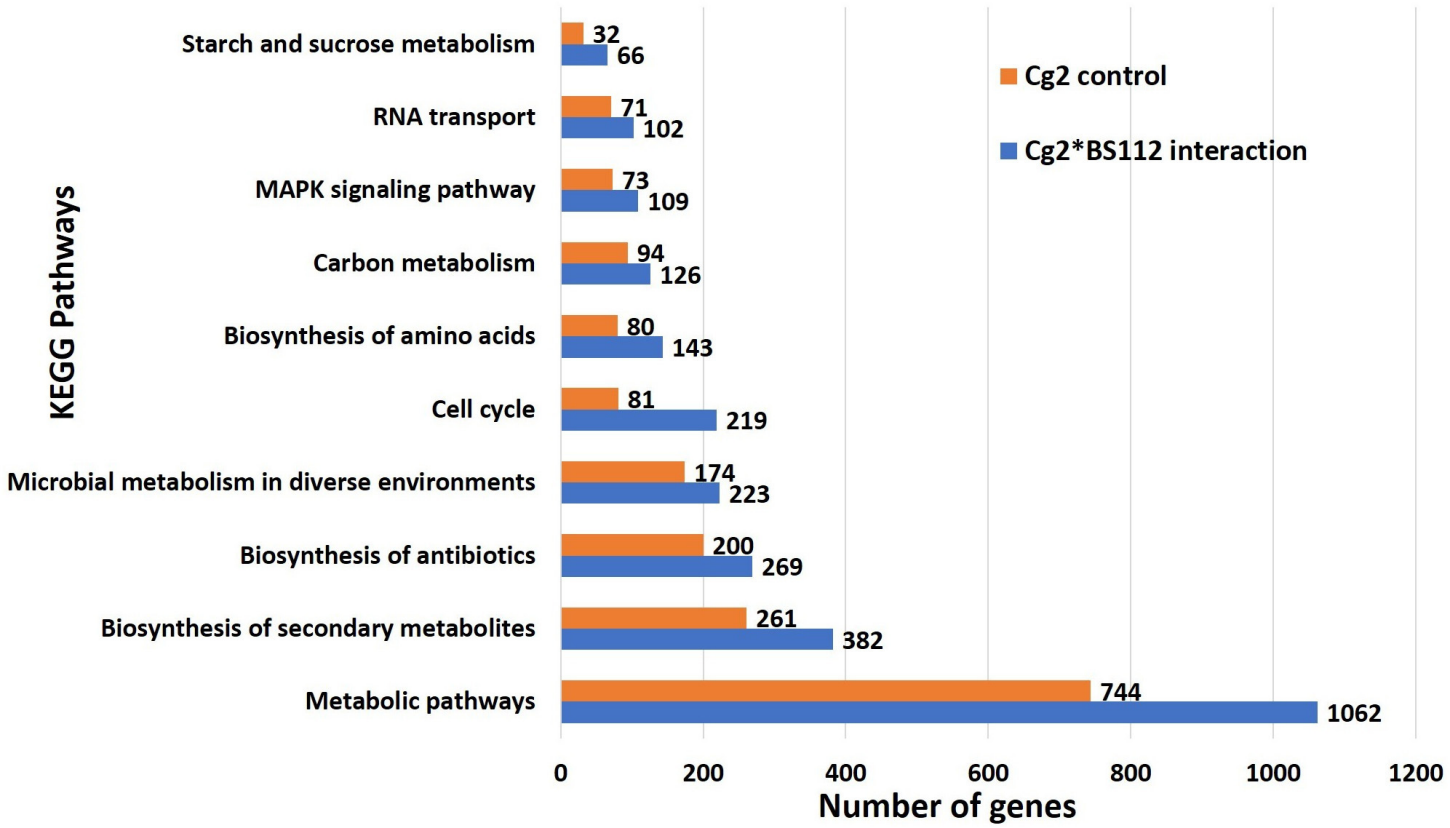
Top 10 significant KEGG pathways identified in *C. globosum* transcripts in response to BS112.

**Figure 6:**
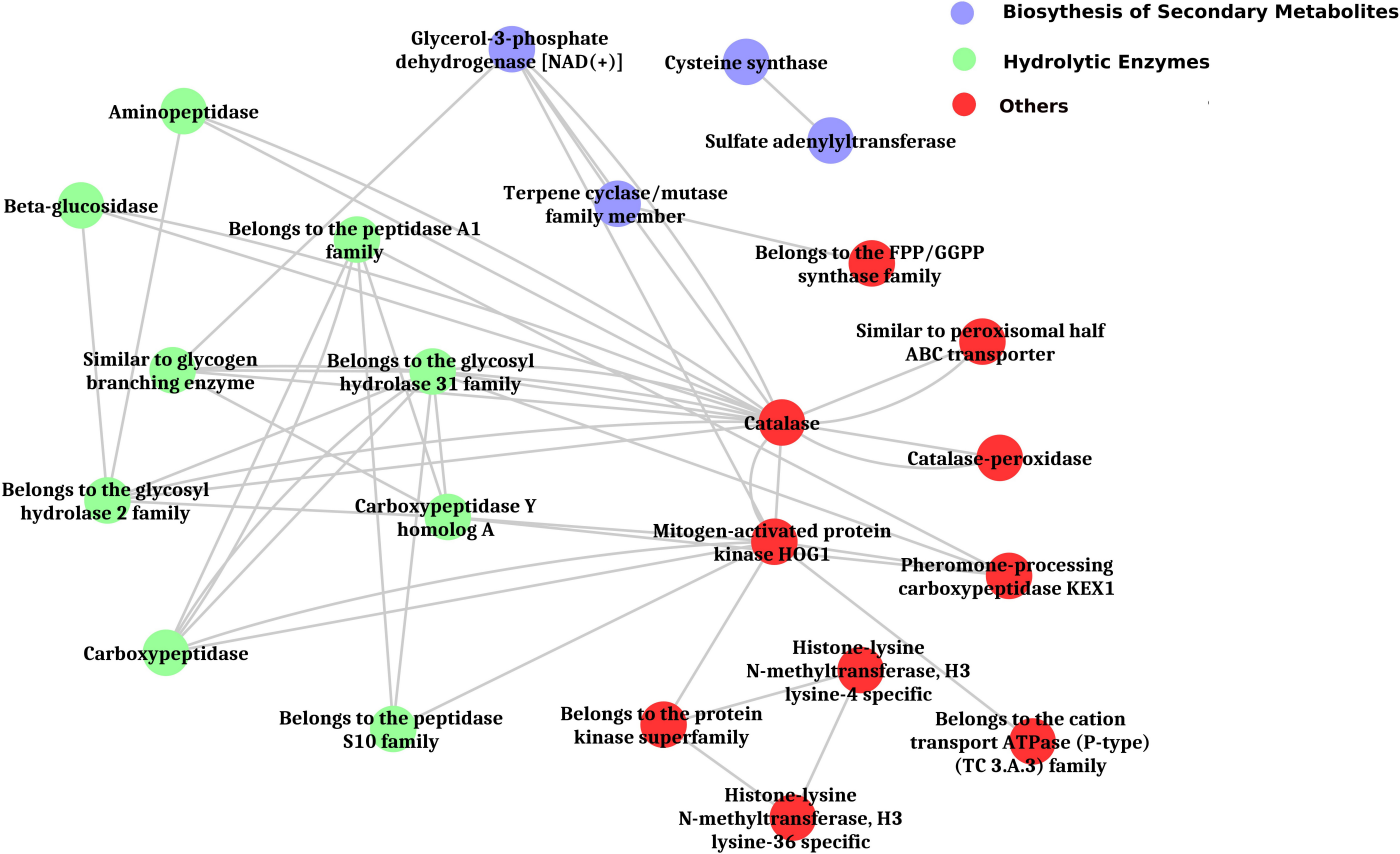
Layout of significantly enriched biological processes and key regulatory genes in Cg2-BS112 interaction.

The inferred proteins with homologs or orthologs in other organisms have been mapped to conserved biological pathways using the KEGG database. KEGG annotated a total of 6,653 (23.79%) sequences with KO terms. The DEGs were further significantly enriched in 171 KEGG pathways by using the Blast2GO tools, out of which, the top five pathways were metabolic pathways, biosynthesis of secondary metabolite, biosynthesis of antibiotics, microbial metabolism in diverse environments, and cell cycle (Figure 5, Supplementary Figure 8, Supplementary Tables 9, 10). Further, filtration of DEGs under different categories such as the genes involved in cell wall degradation, biosynthesis of secondary metabolites, antibiotic biosynthesis genes, transporter genes, transcription factors, and other related genes (lectin, siderophores, and sulfur metabolism), which may support the antagonism of *C. globosum* against *B. sorokiniana*, is listed in Table 2. To know the functional network, the enriched pathways were mapped and visualized using Cytoscape v8.1. The layout depicts the highly expressed genes such as pathways related to biosynthesis of secondary metabolites, hydrolytic enzymes, and other key regulator genes in the Cg2–BS112 interaction (Figure 6 and Supplementary Figures 9A–C).

**Table 2:** Filtration of differentially expressed antagonistic related gene (s) of *C*. *globosum*.

**S. No**	**Gene IDs**	**Protein name**	**log2 fold change**	**Gene ontology**
**Genes involved in cell wall degradation**				
1	XP_962211.3 XP_003667046.1 XP_003658976.1 XP_003656039.1 XP_003650462.1	CAZy category enzymes:Glycosyl hydrolase (GH),GH 13, GH 2, GH 31 and GH family 81 protein	14.00 12.90 7.72 8.78 7.60	Hydrolase activity (GO:0005975); [GO:0004553], 1,4-alpha-glucan branching enzyme activity [GO:0003844), beta-galactosidase activity (GO:0004565), Glucan endo-1,3-beta-glucanase activity [GO:0052861]; carbohydrate binding [GO:0030246]
2	KXX74005.1 XP_001220280.1	Glucan 1,3-beta-glucosidaseBeta-glucosidase (EC 3.2.1.21)	8.60	Beta-glucosidase activity [GO:0008422]; scopolin beta-glucosidase activity [GO:0102483]; cellulose catabolic process [GO:0030245]
3	XP_001228734.1 XP_003664155.1 XP_001229775.1 XP_001230164.1	Aminopeptidase (EC 3.4.11.-); AMP_N domain-containing protein; Peptidase_M24 domain-containing protein; Peptidase A1 domain-containing protein	10.94 9.11 14.44	Aminopeptidase activity [GO:0004177]; metallopeptidase activity [GO:0008237]; zinc ion binding [GO:0008270]; manganese ion binding [GO:0030145]; aspartic-type endopeptidase activity [GO:0004190]
4	XP_001229654.1	Carboxypeptidase Y homolog A (EC 3.4.16.5)	14.66	Serine-type carboxypeptidase activity [GO:0004185].
5	XP_001225331.1	Cellulase domain-containing protein	12.37	Hydrolase activity, hydrolyzing O-glycosyl compounds [GO:0004553]; carbohydrate metabolic process [GO:0005975]
**Genes involved in biosynthesis of secondary metabolites**				
1	XP_003656646.1	Alkaloids (Aminotran_1_2 domain-containing protein)	8.56	Biosynthetic process [GO:0009058]
2	XP_003650550.1 XP_003660598.1	Triterpenoid;Terpene cyclase (EC 4.2.3.-)Squalene epoxidase (EC 1.14.13.132)	4.52	Terpene synthase activity [GO:0010333]Sterol biosynthetic process [GO:0016126]Squalene monooxygenase activity [GO:0004506]
3	KND89597.1	Putative steroid-binding protein 3	3.95	Steroid metabolic process [GO:0008202]
4	XP_001220383.1	Glycerol-3-phosphate dehydrogenase [NAD (+)] (EC 1.1.1.8)	8.87	Biosynthesis of secondary metabolites; glycerophospholipid metabolism; MAPK signaling pathway—yeast
5	XP_003654287.1	Uncharacterized protein	10.38	Biosynthesis of secondary metabolites; terpenoid backbone biosynthesis
6	XP_001227189.1	2-methoxy-6-polyprenyl-1,4-benzoquinol methylase; Ubiquinone biosynthesis methyltransferase COQ5	8.20	Metabolic pathways; biosynthesis of secondary metabolites; ubiquinone and other terpenoid-quinone biosynthesis [GO:0006744]; 2-octaprenyl-6-methoxy-1,4-benzoquinone methylase activity [GO:0043333]
**Antibiotic biosythesis genes**				
	XP_003664878.1	Polyketide synthase	9.72	Biosynthetic process [GO:0009058]; required for production of antifungal polyketide like chaetoglobosin, chaetoviridin and chaetomugilin biosynthesis
	XP_003648799.1	NRPS non ribosomal peptides (NRPs) synthase: S-hydroxymethyl glutathione dehydrogenase (EC 1.1.1.284)	9.61	Metabolic pathways; biosynthesis of secondary metabolites; microbial metabolism in diverse environments; biosynthesis of antibiotics; carbon metabolism;ethanol oxidation [GO:0006069]
	XP_003663237.1	Histone-lysine N-methyltransferase (EC 2.1.1.43)	8.65	Lysine degradation
	AEQ54765.1 XP_003663032.1	Catalase-peroxidase (CP)(Peroxidase/catalase)	8.74	Metabolic pathways; biosynthesis of secondary metabolites; phenylalanine; tryptophan metabolism; phenylpropanoid biosynthesis; catalase activity [GO:0004096]
	XP_003656646.1	Aminotran_1_2 domain-containing protein	8.60	Biosynthetic process [GO:0009058]; biosynthesis of antibiotics; 2-oxocarboxylic acid metabolism; biosynthesis of amino acids; catalytic activity [GO:0003824)
**Transporter genes**				
	XP_003655213.1 XP_003658468.1 XP_003662561.1 XP_0012297671 KXX77612.1 XP_003655240.1	ABC transporter	14.30 11.00	Drug transmembrane transport [GO:0006855]; export across plasma membrane [GO:0140115]; fatty-acyl-coA transport [GO:0015916]; integral component of membrane [GO:0016021]; plasma membrane [GO:0005886]; ATPase-coupled transmembrane transporter activity [GO:0042626]
	XP_003658958.1 XP_003660898.1 XP_001220779.1 OPB37661.1 KXX78384.1	Amino acid permease domain-containing protein;Purine-cytosine permease fcyB;Amino-acid permease;MFS permease; Amino-acid permease BAT1	8.72	Transmembrane transporter activity [GO:0022857]; integral component of membrane [GO:0016021]; amino acid transport [GO:0006865]; transmembrane transport [GO:0055085]
	XP_003665357.1 XP_003663203.1 XP_003660547.1	MFS domain-containing protein	13.33 13.32 12.91	Integral component of membrane [GO:0016021]; transmembrane transport [GO:0055085]
**Transcription factors**				
	KXX77270.1 XP_001229428.1 XP_003664212.1	Cross-pathway control protein 1;BZIP domain-containing protein;NDT80 domain-containing protein	18.21 11.48 12.26	DNA-binding transcription factor activity [GO:0003700]: potential StzA binding sites were identified in cpcA (cross pathway control regulator of amino acid biosynthesis) and it intriguing link between intracellular amino acid availability and cellulase gene expression
	XP_003664314.1 XP_001912977.1 XP_003662981.1	Copper-fist domain-containing protein; Podospora anserina S mat+ genomic DNA chromosome 1, supercontig 1; STE like transcription factor	13.41 13.56 8.66	Nucleus [GO:0005634]; copper ion binding [GO:0005507]; DNA binding [GO:0003677]; DNA-binding transcription factor activity [GO:0003700]; nucleic acid binding [GO:0003676]
	XP_001219949.1 XP_003667226.1 XP_001220814.1	Zn (2)-C6 fungal-type domain-containing protein; Zinc finger protein klf1; C6 finger domain-containing protein	13.01 13.00 12.98	Nucleus [GO:0005634]; DNA binding [GO:0003677]; DNA-binding transcription factor activity, RNA polymerase II-specific [GO:0000981]; zinc ion binding [GO:0008270]; transcription, DNA-templated [GO:0006351]
	XP_003661209.1 XP_003660612.1	Fork-head domain-containing proteinC2H2 transcription factor	10.52 7.13	Nucleus [GO:0005634]; DNA-binding transcription factor activity [GO:0003700]; sequence-specific DNA binding [GO:0043565]; regulation of transcription, DNA-templated [GO:0006355]: It essential for starch degradation and a regulator of glucose signaling and metabolism.
	XP_003665535.1	Protein kinase domain-containing protein	11.21	Dbf4-dependent protein kinase complex [GO:0031431]; nucleus [GO:0005634]; ATP binding [GO:0005524]; protein serine/threonine kinase activity [GO:0004674]; G1/S transition of mitotic cell cycle [GO:0000082]; mitotic DNA replication checkpoint [GO:0033314]; mitotic DNA replication initiation [GO:1902975]; negative regulation of DNA binding [GO:0043392]; negative regulation of transcription by transcription factor catabolism [GO:0010620]; replication fork protection [GO:0048478]; signal transduction involved in intra-S DNA damage checkpoint [GO:0072428]
**Other antagonistic related genes**				
	XP_003655678.1 XP_003665470.1	**Lectin**: L-type lectin-like domain-containing protein; Jacalin-type lectin domain-containing protein	7.23	Protein processing in endoplasmic reticulum; integral component of membrane [GO:0016021]; Role mediating the physical contact and elicitation of the signaling cascadeG-proteins and MAPKs
	CCO36989.1	**Siderophores**: Uncharacterized protein	7.82	Siderophore biosynthetic process [GO:0019290]
	KXX82629.1 XP_001227181.1 XP_001221807.1	**Sulphur metabolism**:Sulfate adenylyltransferase (EC 2.7.7.4) (ATP-sulfurylase) (Sulfate adenylate transferase) (SAT);Cysteine synthase	11.06 12.58	Cytoplasm [GO:0005737]; adenylyl sulfate kinase activity [GO:0004020]; ATP binding [GO:0005524]; sulfate adenylyl transferase (ATP) activity [GO:0004781]; cysteine biosynthetic process [GO:0019344]; hydrogen sulfide biosynthetic process [GO:0070814]; methionine biosynthetic process [GO:0009086]; sulfate assimilation [GO:0000103]cysteine biosynthetic process from serine [GO:0006535]

### Validation of Prominent Antagonistic Related Genes Through qRT-PCR

**Figure 7:**
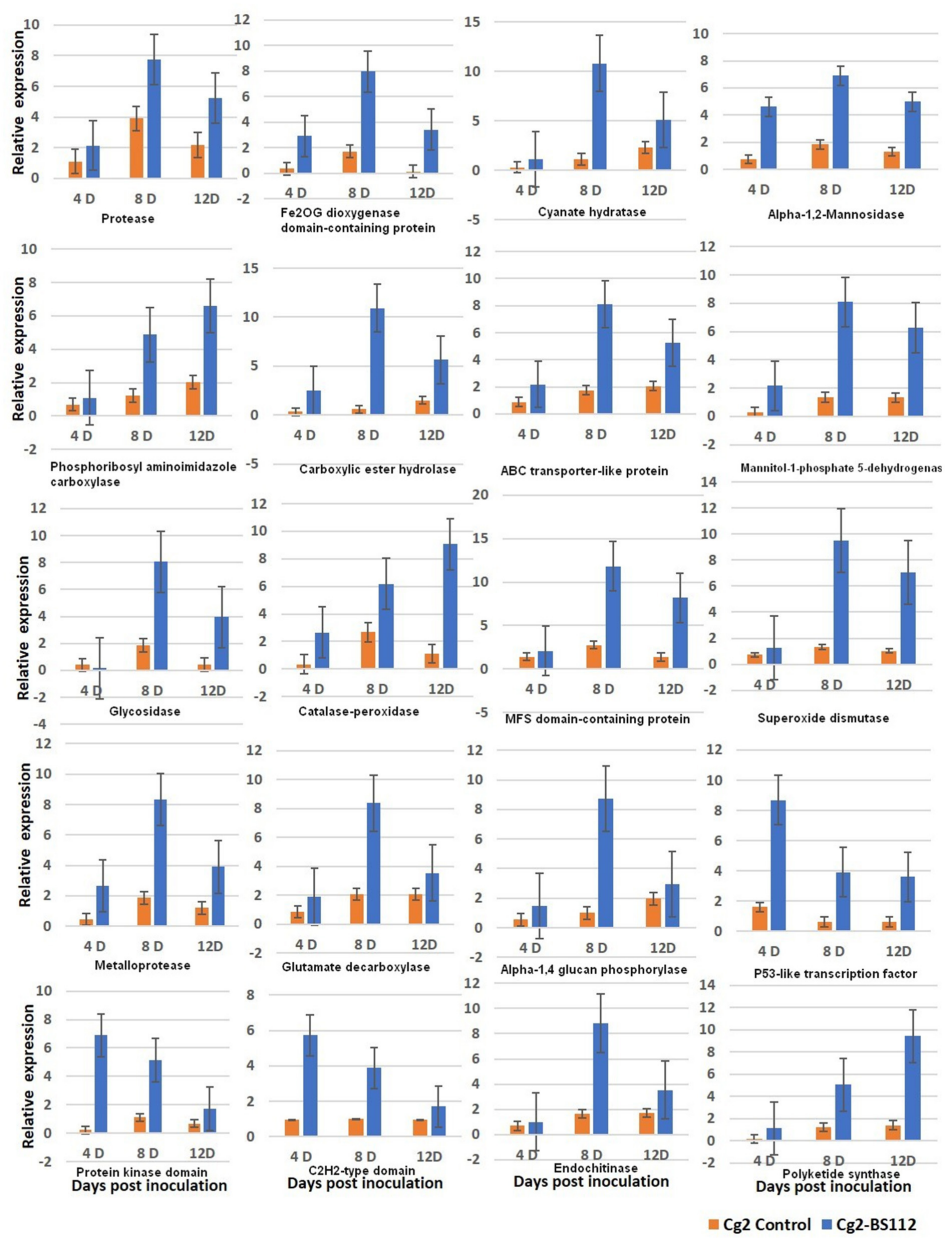
qRT-PCR validation of selected genes showed significant difference in their expression in Cg2-BS112 interaction when compared with Cg2 alone at three different time intervals. Error bars shows ±SD among the biological triplicates.

qRT-PCR was performed for 20 selected genes to check the reliability of RNA-seq data obtained by *in silico* analysis. The RNA was isolated at day 3 because it is assumed that genetic effects leading to the observed physical differences at day 6 are expected to be initiated earlier, taking into account the time needed to produce, secrete, and accumulate metabolites to considerable levels that are sufficient to show the antagonistic mechanism (Kosawang et al., 2014). To understand the expression kinetics in a better way, three time points were maintained (3D, 5D, and 9D). The patterns of gene expression in the interaction were analyzed by comparing their expressions in control at three time points (initial, middle, and final). The tested genes exhibited a similar level of expression in the RT-qPCR and RNA-seq analyses. Overall, the obtained fold change profiles of 20 tested genes from qRT-PCR fully agreed with those of RNA-seq expression analysis and confirmed the patterns of expression of the genes identified in the RNA-seq analysis (Figure 7, Supplementary Figure 10, and Supplementary Table 11).

## Discussion

*C. globosum* has been reported to be a potential biocontrol agent against various soilborne and seedborne phytopathogens (Aggarwal et al., 2004; Aggarwal, 2015; Moya et al., 2016; Jiang et al., 2017). In general, it has been found to be effective through exerting multiple mechanisms including mycoparasitism, antibiosis, and competition (Aggarwal et al., 2004; Moya et al., 2016; Zhao et al., 2017; Yue et al., 2018). The selection of the most effective biocontrol strains is primarily associated with understanding the mechanism(s) of their action involved in the biocontrol process with the pathogens, and that provides information about where and when the interaction occurs and how the pathogens will be affected by interferences in its life process (Larkin et al., 1996; Aggarwal, 2015). Therefore, in recent years, advances in genomics have discovered a large number of enzymes, signal molecules, transcription factors, secondary metabolites, and new metabolic pathways. It is now possible to understand why some strains can control the growth of a given pathogen more effectively than others by employing various omics technology. To date, the most well-studied fungi include many strains of the genera *Trichoderma, Clonostachys rosea*, and *Coniothyrium minitans*, but the mechanisms underlying this connection in *C. globosum* have not been studied so far. Keeping this in view, we performed a high-throughput transcriptome sequencing to identify the antagonistic related genes expressed during the interaction of *C. globosum* with the pathogen *B. sorokiniana*, and DEGs during interactions were annotated, and a subset of such differentially regulated genes during antagonisms were verified by qRT-PCR.

Differential gene expression analysis revealed a total of 14,366 genes which were significantly DEGs in *C. globosum* Cg2 during challenge with *B. sorokiniana* BS112. Among them, 6,109 were found to be unique DEGs for the Cg2–BS112 interaction. These unique DEGs depict their dynamic differences in the Cg2–BS112 interaction and Cg2 control that can be related to precontact and antagonistic interactions with *B. sorokiniana*. A similar study conducted in *Rhizoctonia solani, Serratia proteamaculans*, and *Serratia plymuthica* interaction resulted in a dynamic expression of 462 and 242 DEGs (Gkarmiri et al., 2015). Understanding the mechanism(s) of action involved in the biocontrol process is of primary importance in determining the potential of biocontrol agents and will contribute significantly to knowing how the pathogens will be affected (Mukherjee et al., 2014; Aggarwal, 2015). Of note, the genes assigned to the functionality group “metabolic pathways” and “metabolic process” represented the majority of genes that were upregulated during interactions with *B. sorokiniana*. Such findings lead to the assumption that this biocontrol agent likely improves the availability of nutrients by host fungus cell wall lysis, which further triggers the growth and fitness of *C. globosum*. The results presented in this study support our hypothesis that when *C. globosum* is confronted with *B. sorokiniana*, the predominant expression of transcripts associated with “catalytic activity” constituted 45.06%, out of which 10.02% were involved in “hydrolytic activity” (GO:0008152) under MFs and, similarly in the BP, 29.18% were involved in “metabolic activity” (GO:0004096 and GO:0006979), which were found to be differentially upregulated. We interpret the genes in the main gene ontology categories, hydrolase activity (GO:0005975), peptidase activity (GO:0004177), biosynthesis of secondary metabolites (GO:0006069), biosynthesis of antibiotics (GO:0009058), and transmembrane transporter activity (GO:0022857), which suggested a central role for them in *C. globosum* antagonisms, probably during host fungal cell wall degradation and mycelial inhibition. Similar studies were reported earlier in the antagonistic activity of *Trichoderma* strains synergistically producing cell wall-degrading enzymes (CWDEs) followed by production of metabolites with antibiotic functions against fungi and bacteria (Reithner et al., 2011; Vorapreeda et al., 2015). Liu et al. (2019) applied transcriptomic approaches to explore the novel biocontrol mechanisms of *Bacillus amyloliquefaciens* and *Bacillus subtilis* against *Phytophthora* blight of soybean (*Phytophthora sojae*). In the analysis, the upregulated GO terms were the threonine-type endopeptidase activity, hydrolase activity, biosynthetic process, and proteolysis involved in mycelial degradation and protein catabolic process.

In agreement with the above assumption, all the genes involved in fungal cell wall degradation were reported to be significantly upregulated in this study. Interestingly, the genes for CAZYmes such as the GH family (GH 13, GH 2, GH 31, and GH 81) (hydrolase activity [GO:0005975]), cellulase domain-containing protein, chitinases, β-1-3-glucanase (GO:0004565), and carboxylic ester hydrolase (GO:0016787) were differentially expressed in the Cg2–BS112 interaction, which act mainly as fungal CWDEs. Similar results on upregulation of CWDEs have been recorded by Atanasova et al. (2013), suggesting a central role in mycoparasitism in *Trichoderma atroviride*. Aggarwal (2015) studied the mechanism of biocontrol by cell wall degradation incited by beta-glucanases and carboxymethyl cellulases of *C. globosum* against *B. sorokiniana*, which were found to play an important role in cell wall degradation. An extracellular β-1,3-glucanase produced by *C. globosum* (Cg2) was purified and characterized by Ahammed et al. (2012). A gene of *C. globosum*, 46 kDa, codes for an endochitinase (*Chi*46) that degrades the cell walls of fungal pathogens, e.g., *R. solani, Fusarium oxysporum, Sclerotinia sclerotiorum, Valsa sordida, Septoria tritici*, and *P. sojae* (Liu et al., 2008). The genes encoding secreted proteases such as aminopeptidase (GO:0004177), metallopeptidase (GO:0008237), aspartic-type endopeptidase (GO:0004190), and serine-type carboxypeptidase (GO:0004185) were expressed significantly and involved in the degradation of fungal cell walls and plasma membrane proteins. They are required for the complete hydrolysis of pathogenic cell walls (Liu et al., 2014). Wu et al. (2017) reported similar results through transcriptomic approaches in *Trichoderma asperellum* GDFS1009. This process is accompanied by the secretion of CWDEs that penetrate the pathogenic mycelium, absorbing its nutrients and eventually dissolving the pathogen. Steindorff et al. (2014) reported the same pattern of expression in the interaction of *Trichoderma harzianum* strain TR274 against *S. sclerotiorum* using Illumina sequencing. The expression studies have shown that several genes of biological value, encoding proteins with functions such as hydrolytic activity (CAZYmes, GH 18 and GH 25, chitinases, α-1,3-glucanase, and proteases/peptidases), were upregulated to degrade the host cell wall. These results strongly suggest a role of these enzymes in the mycoparasitism of *C. globosum* against *B. sorokiniana*. Mycoparasitism was observed in the interaction between *C. globosum* and *B. sorokiniana*, along with a reduction of the pathogen mycelium growth in the present study. The mode of action in terms of direct interaction including the production of hydrolytic CWDEs as well as biocidal substances is based on a mycotrophic lifestyle of the antagonistic organisms (Qualhato et al., 2013; Khatri et al., 2017), which induces strong hydrolysis of pathogenic cell walls and results in inhibition of spore germination and mycelial digestion (Sun et al., 2005). A similar mechanism of inhibition was reported in *Trichoderma* spp. against various fungal plant pathogens like *R. solani, Sclerotinia sclerotiorium, Alternaria alternata, Botrytis cinerea*, and *Fusarium* spp. (Geistlinger et al., 2015; Juliatti et al., 2019).

Antibiosis was the most prevalent antagonistic mechanism of *C. globosum* against *B. sorokiniana*, in agreement with studies by Aggarwal et al. (2004). Mandal et al. (1999) and Biswas et al. (2000) also observed this mechanism in their experiments. Istifadah and McGee (2006) found the same effect against *Drechslera tritici-repentis*. *C. globosum* induces pathogen inhibition by secreting secondary metabolites. *Chaetomium* spp. produces a variety of bioactive metabolites such as chaetoglobosins A and C, chaetomin, cochliodinol, chaetosin, chaetomugilins D and A, and prenisatin (Aggarwal et al., 2011, 2013; Zhang et al., 2013), which suppress the growth of many soilborne and seedborne phytopathogens. In the present study, the higher expression of genes related to polyketide synthase (biosynthetic process [GO:0009058]) was observed in the interaction, which is required for production of antifungal polyketides like chaetoglobosin, chaetoviridin, and chaetomugilin (Yang et al., 2012). In addition, alkaloids (aminotran_1_2 domain-containing protein), triterpenoid (terpene cyclase and terpene synthase activity [GO:0010333]), *S*-hydroxymethyl glutathione dehydrogenase (biosynthesis of antibiotics [GO:0006069]), and methoxy-polyprenyl benzoquinol methylase (biosynthesis of secondary metabolites; ubiquinone; and other terpenoid-quinone biosynthesis [GO:0006744]) were expressed significantly in *C. globosum* Cg2 when exposed to *B. sorokiniana* and are in agreement with the results of validation by RT-qPCR. Similar unigenes participating in the terpenoid backbone biosynthesis were also found in *Trichoderma brevicompactum* by Shentu et al. (2014). Qin et al. (2009) reported novel cytotoxic chlorinated azaphilone derivatives, viz., chaetomugilins D and A and chaetoglobosins A and C, which were isolated by a bioassay-guided fractionation from the EtOAc extract of *C. globosum* that showed antifungal activity by inhibiting the spore germination of *Artemia salina* and *Mucor miehei*. Similarly, Aggarwal et al. (2013) have characterized the antifungal metabolites of *C. globosum* and its antagonist against fungal plant pathogens. Chaetoglobosin A and cochliodinol from *C. globosum* have shown antifungal activity. A previous proteome study in *Chaetomium* spp. (Sharma et al., 2014) supported our findings. They identified proteins, of which 79% were hypothetical proteins and 21% were functionally characterized proteins such as polyketide synthase, putative GTP binding protein, endochitinase, β-1,3-glucanase, polyendogalactouronase, *hsp*22, and mitogen-activated protein kinase, which are involved in different biocontrol activities like cell wall degradation, stress tolerance, and antagonistic activity against fungal plant pathogens.

*C. globosum* mycoparasitizes and produces antifungal metabolites that help in lysis of the pathogen cell wall. The lysed cell wall was utilized as carbon and nitrogen sources as a nutrient for its growth and development. On the other hand, *Trichoderma* can also use the lysed fungal cell wall as a source of carbon and nitrogen (Ait-Lahsen et al., 2001). In our results, cyanate hydratase (D1ZHN6.2) is an enzyme involved in the metabolism of nitrogen, which catalyzes the reaction of cyanate with bicarbonate to the production of ammonia and carbon dioxide. The cyanate-generated ammonium can serve as a source of nitrogen for *C. globosum* growth (Walsh et al., 2000). A fungal transcription factor, C_2_H_2_-type domain-containing protein, has been upregulated in the Cg2–BS112 interaction, suggesting an essential role in starch degradation and a regulator of glucose signaling and metabolism. Fork-head domain-containing protein, a C_2_H_2_ transcription factor, was expressed and involved in the carbohydrate metabolic process (GO:0005975) and closely related to the utilization of carbohydrates and energy production, resulting in the inhibition of *B. sorokiniana* growth. A similar investigation on the antagonism by competition for nutrients, which limited the growth of *B. sorokiniana* and *Drechslera teres* pathogens by *C. globosum*, was performed earlier (Vannacci and Harman, 1987; Moya et al., 2016). Competition plays another important role in the suppression of soilborne and foliar pathogens (Howell, 2003; Harman et al., 2004). In the present study, out of the 213 expressed genes, 98 encode MFS transporter proteins and 19 encode ABC transporter proteins. The MFS transporters are the most abundant proteins in the Cg2–BS112 interaction between the transporter proteins. These proteins make it possible to carry essential nutrients, ions, and metabolic products. Furthermore, the energy of adenosine triphosphate binding is used to perform BPs such as excretion of hydrolytic enzymes and secondary metabolites to extracellular environments across the membranes (Davidson et al., 2008).

Studies of genome-wide expression are valuable methods for detecting genes associated with different BPs, such as mycoparasitism and antibiosis. The information obtained allows for the selection of potential candidate genes for functional characterization to gain in-depth knowledge on the physiological processes taking place in the presence of host fungi. Our findings showed the global antagonistic mechanisms of *C. globosum* Cg2 at the molecular level, including biocontrol factors related to mycoparasitism and antibiosis using transcriptomic analysis. Further studies for functional characterization of candidate genes mentioned here are necessary in order to better define the exact pathways involved in mycoparasitism and antibiosis in *C. globosum*. The comprehensive gene expression profile will provide a blueprint for further understanding of biocontrol mechanism of this fungus.

## Conclusion

In this study, we obtained the transcriptome information of *C. globosum*, including functional annotation and classification, which could provide insight into the role of intracellular metabolic pathways. Illumina HiSeq 2000 sequence platform generated an average of 19–23 million short reads, which were assembled into 55,173 transcripts in *C. globosum*. In summary, the 27,957 unigenes showed a match with the GO annotation. Meanwhile, biosynthetic pathways involving some of these unigenes have also been identified. Based on these findings, we infer that several mycoparasitism-related enzymes, secondary metabolites, and antibiosis-related genes were involved in *C. globosum* but have not yet been explored, including chitinases, proteases, polyketides, and antimicrobial peptides. Mycoparasitism and antibiosis have been proposed as the most effective machinery exhibited by *C. globosum* against *B. sorokiniana*. In the global context, the present work is the first to report on the role of *C. globosum* antagonism against *B. sorokiniana*, which facilitates the improvement of gene model annotation in the *C. globosum* draft genome. The obtained data will greatly enrich current *C. globosum* genetic information and provide a good foundation for further studies on *C. globosum* to facilitate widespread application in the field of biocontrol in agriculture.

## Data Availability Statement

The datasets generated for this study can be found in online repositories. The names of the repository/repositories and accession number(s) can be found below: https://www.ncbi.nlm.nih.gov/, SRR11305503; SRR11305502; SRR113055501; SRR113055500; SRR11305499; and SRR11305498.

## Author Contributions

KD, RA, BB, and VS were involved in the conceptualization of the project, study design, critical inputs, and finalization of the manuscript. KD and JS were involved in wet lab experiments. KD, JS, MG, and AS were involved in bio-informatics analyses and data compilation. KD, BB, and RA have drafted the manuscript. VS, AS, and RA edited the manuscript. All authors have read and approved the final manuscript.

## Conflict of Interest

The authors declare that the research was conducted in the absence of any commercial or financial relationships that could be construed as a potential conflict of interest.

## Supplementary Material

Supplementary Figure 1 Co-incubation of *C. globosum* (Green arrow) and *B. sorokiniana* (Red arrow) for at different intervals. **(A)** 3 DAI, **(B)** 6 DAI, **(C)** 9 DAI. DAI—Days after incubation.

Supplementary Figure 2 Illumina raw data statistics showing good base quality score.

Supplementary Figure 3 Read mapping assembly cleaned reads against reference genome available in NCBI GenBank.

Supplementary Figure 4 Transcript homology distribution of top 10 species with most homologs to *C. globosum*. The distribution was calculated using best BLASTX hit.

Supplementary Figure 5 Graphical representation of significant DEGs through MA plot based on log_2_ fold change value. The red dots signify differentially expressed transcripts.

Supplementary Figure 6 Venn diagram showing the unique (non-overlapping region) and common expressed genes (overlapping region) obtained when *C. globosum* (Cg2) challenged with *B. sorokiniana* (BS112).

Supplementary Figure 7 Pie chart representation of GO terms; biological process **(A)**, molecular function **(B)**, and cellular component **(C)**.

Supplementary Figure 8 Top 10 enriched KEGG pathways of differentially expressed genes in Cg2-BS112 interaction.

Supplementary Figure 9 Metabolic maps show putative pathways in secondary metabolism in *C. globosum* (Cg2) upon challenge with *B. sorokiniana* (BS112). Blue color shows associated genes were upregulated and red color represents downregulated.

Supplementary Figure 10 Melting curves of different genes used for the real time PCR assays.

Supplementary Table 1 RNA Quality and Concentration is estimated using Qubit 4.0 Fluorometer.

Supplementary Table 2 List of validated primers used for Real time PCR.

Supplementary Table 3 Quality check statistics of transcriptomic data by FastQC.

Supplementary Table 4 Assembly statistics of transcriptome generated by Trinity (version 2.4.0) assembler.

Supplementary Table 5 List of Species distribution with most homologs to *Chaetomium globosum*.

Supplementary Table 6 List of differential expressed upregulated and down regulated genes in Cg2-BS112 interaction.

Supplementary Table 7 List of commonly expressed genes in Cg2-BS112 interaction.

Supplementary Table 8 Gene ontology of differentially expressed genes, categorized into three sub categories, i.e., Biological process, molecular function, and cellular component.

Supplementary Table 9 List of KEGG pathways with associated genes in Cg2-BS112 interaction.

Supplementary Table 10 KEGG Pathway enrichment analysis of differentially expressed genes.

Supplementary Table 11 Validation of 20 selected antagonistic related genes through qRT-PCR.
