# Supplementary figures and images for: Transcriptome Profiling Provides Insights Into Potential Antagonistic Mechanisms Involved in Chaetomium globosum Against Bipolaris sorokiniana
Source: Front Microbiol. 2020 Dec 7;11:578115. doi: 10.3389/fmicb.2020.578115 (PMC7750538; doi:10.3389/fmicb.2020.578115)

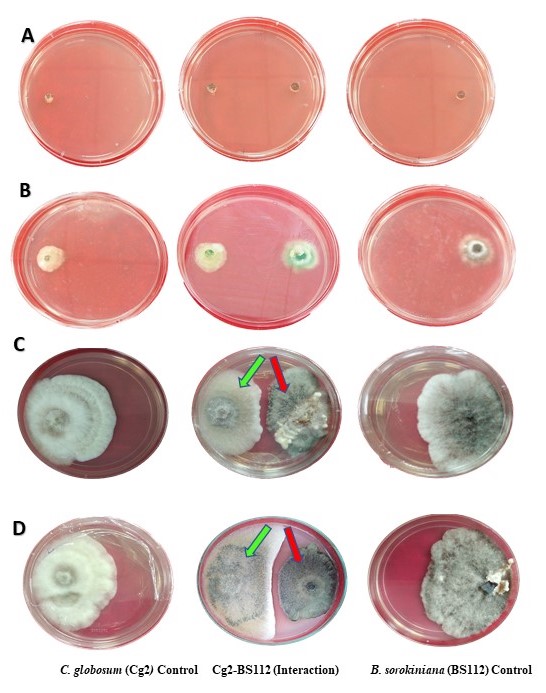

Supplement: Supplementary Figure 1 — Co-incubation of C. globosum (Green arrow) and B. sorokiniana (Red arrow) for at different intervals. (A) 3 DAI, (B) 6 DAI, (C) 9 DAI. DAI—Days after incubation. [file Image_1.JPEG]

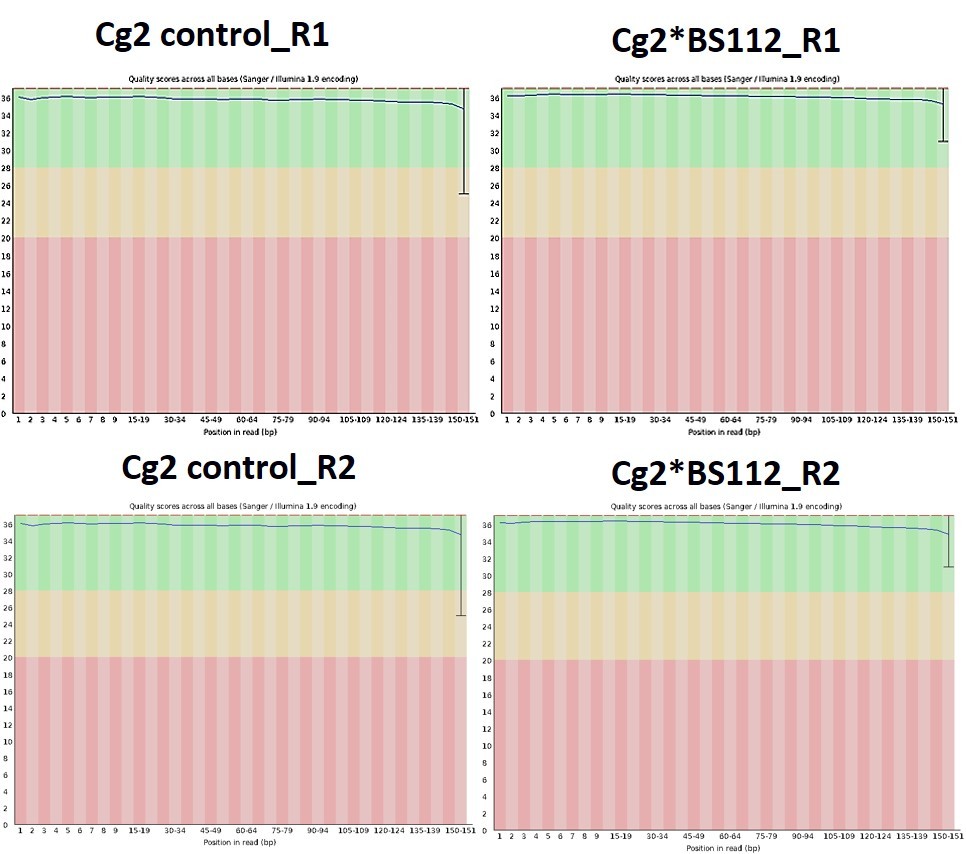

Supplement: Supplementary Figure 2 — Illumina raw data statistics showing good base quality score. [file Image_2.JPEG]

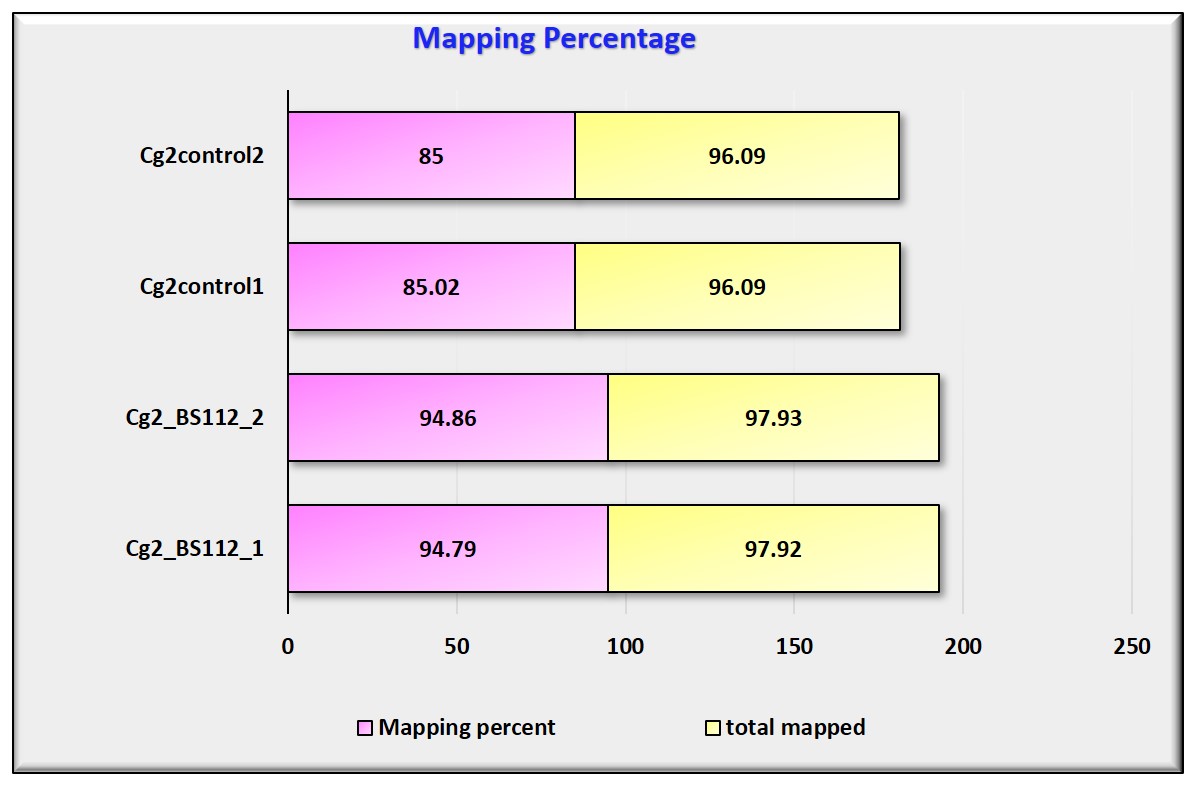

Supplement: Supplementary Figure 3 — Read mapping assembly cleaned reads against reference genome available in NCBI GenBank. [file Image_3.JPEG]

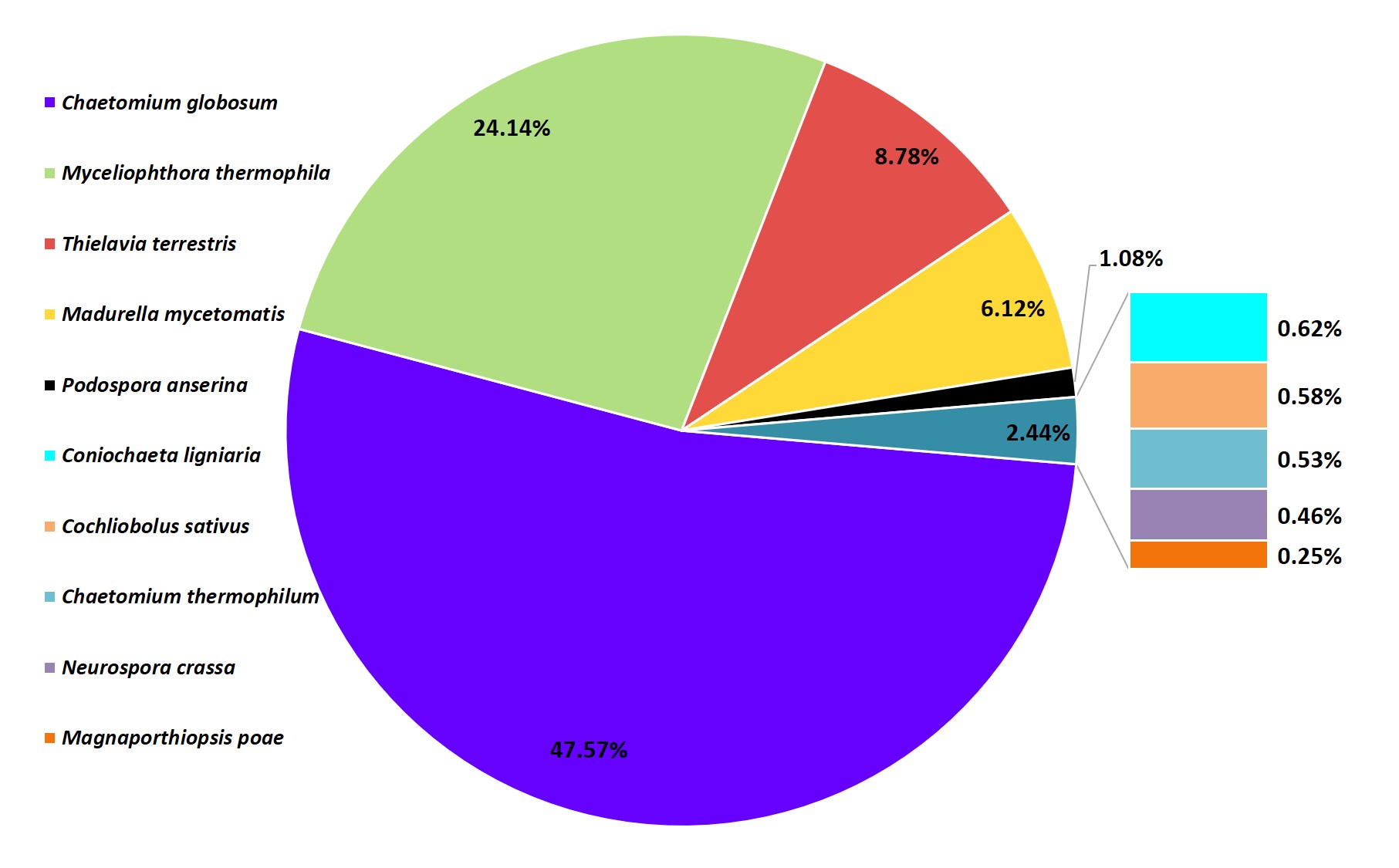

Supplement: Supplementary Figure 4 — Transcript homology distribution of top 10 species with most homologs to C. globosum. The distribution was calculated using best BLASTX hit. [file Image_4.JPEG]

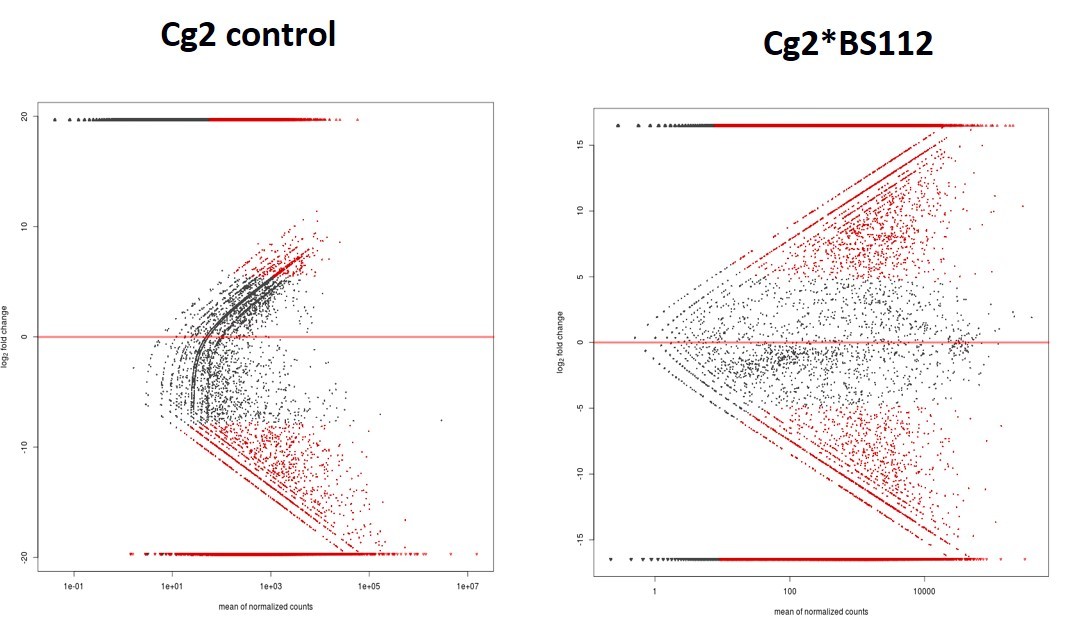

Supplement: Supplementary Figure 5 — Graphical representation of significant DEGs through MA plot based on log2 fold change value. The red dots signify differentially expressed transcripts. [file Image_5.JPEG]

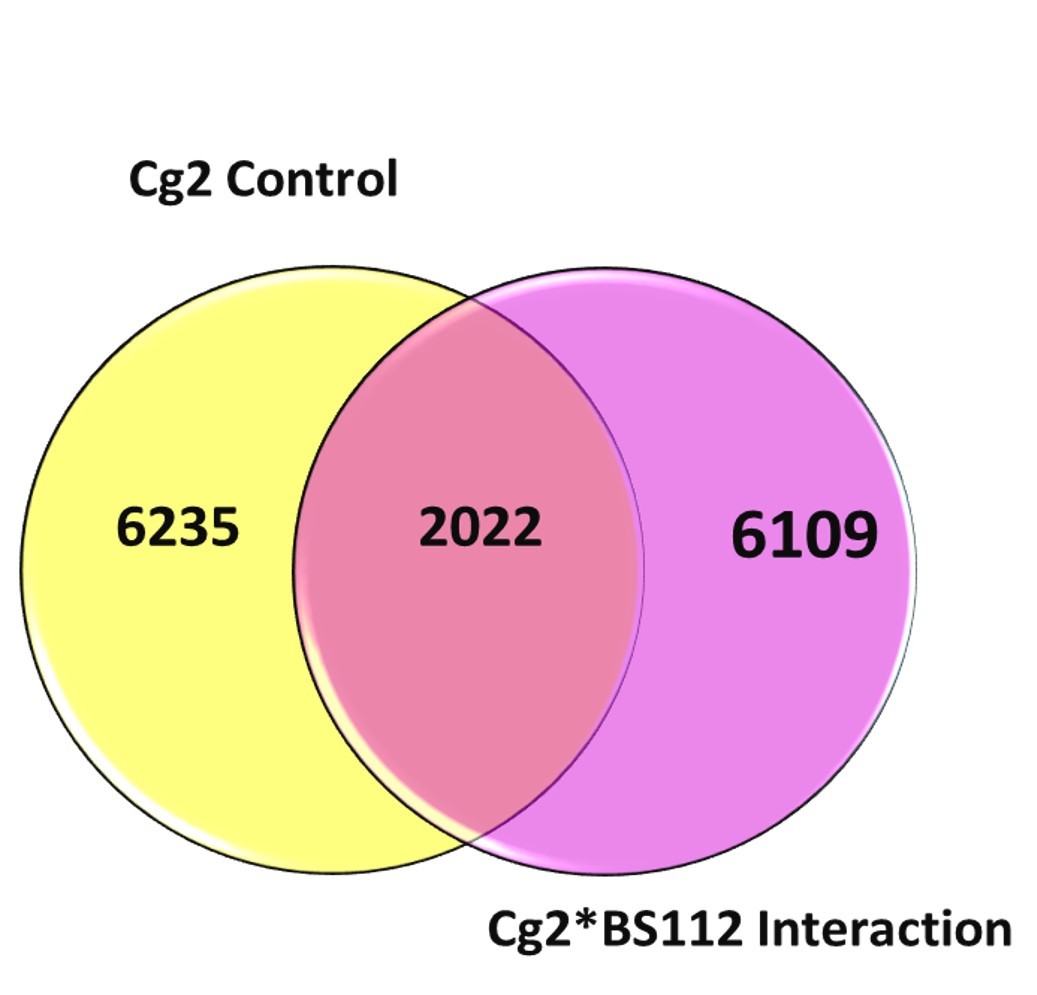

Supplement: Supplementary Figure 6 — Venn diagram showing the unique (non-overlapping region) and common expressed genes (overlapping region) obtained when C. globosum (Cg2) challenged with B. sorokiniana (BS112). [file Image_6.JPEG]

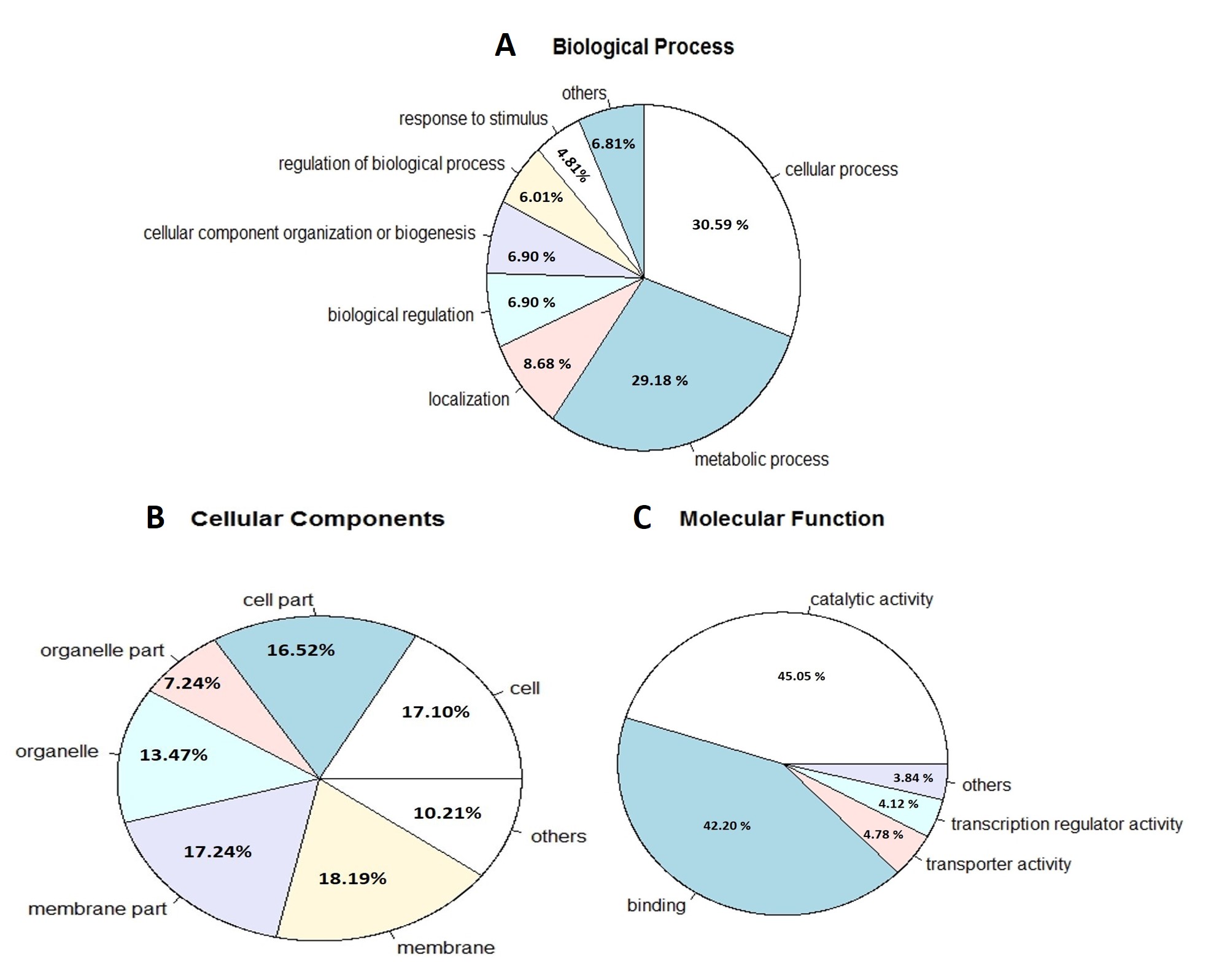

Supplement: Supplementary Figure 7 — Pie chart representation of GO terms; biological process (A), molecular function (B), and cellular component (C). [file Image_7.JPEG]

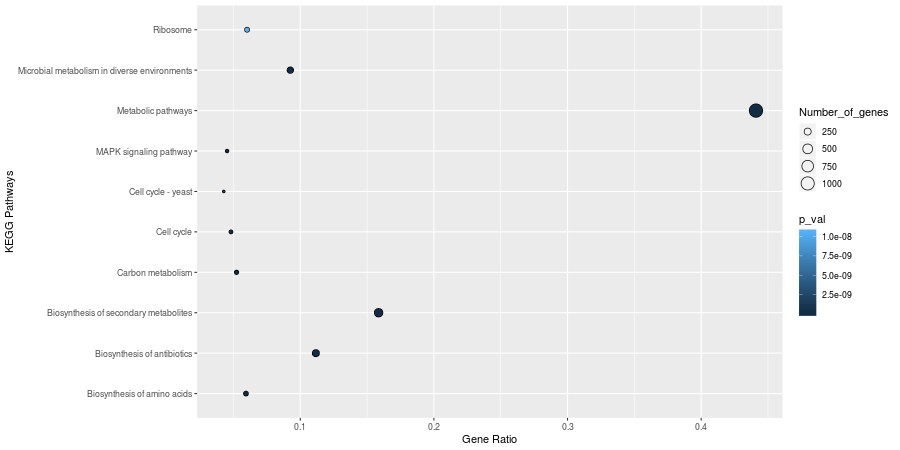

Supplement: Supplementary Figure 8 — Top 10 enriched KEGG pathways of differentially expressed genes in Cg2-BS112 interaction. [file Image_8.JPEG]

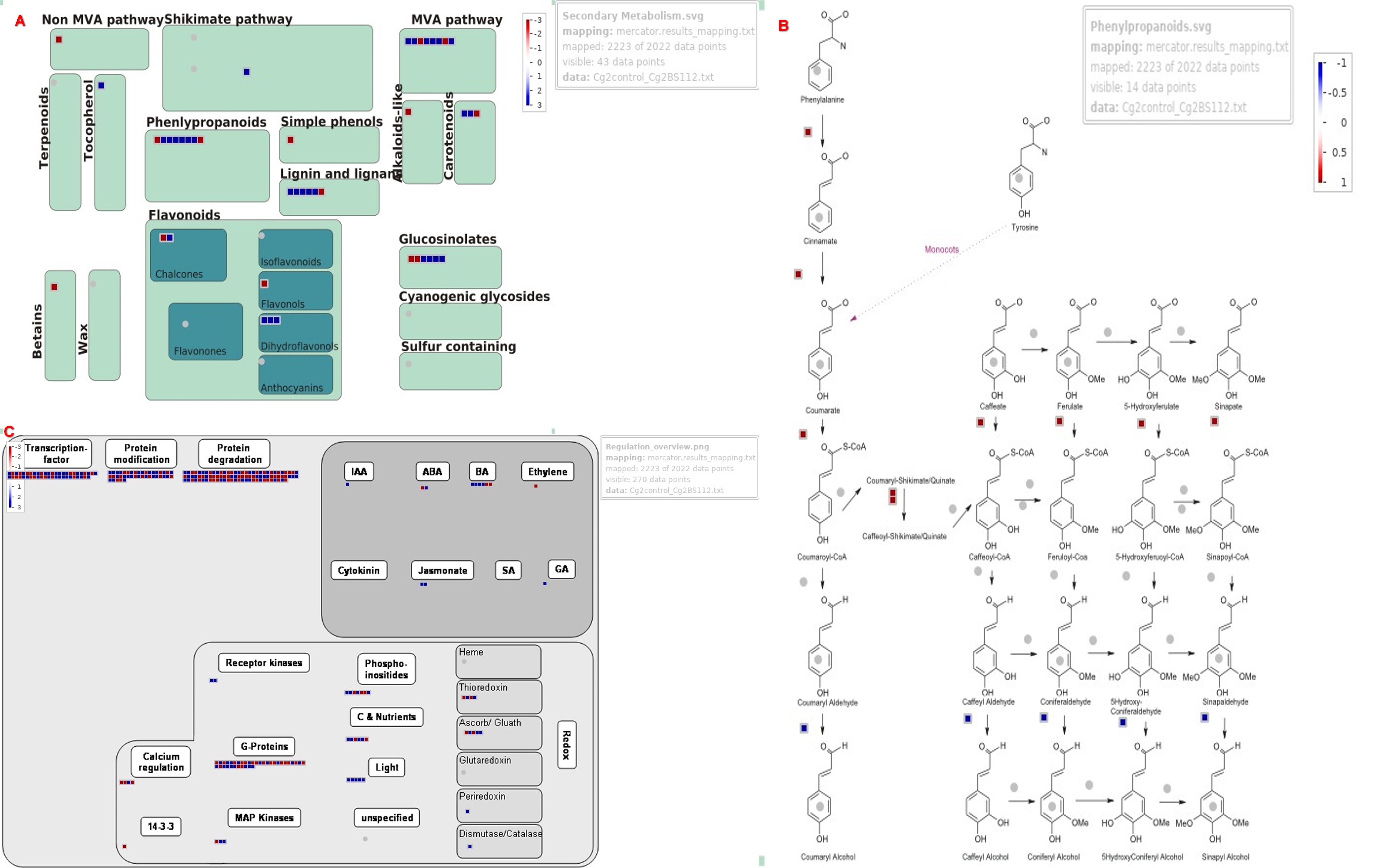

Supplement: Supplementary Figure 9 — Metabolic maps show putative pathways in secondary metabolism in C. globosum (Cg2) upon challenge with B. sorokiniana (BS112). Blue color shows associated genes were upregulated and red color represents downregulated. [file Image_9.jpg]

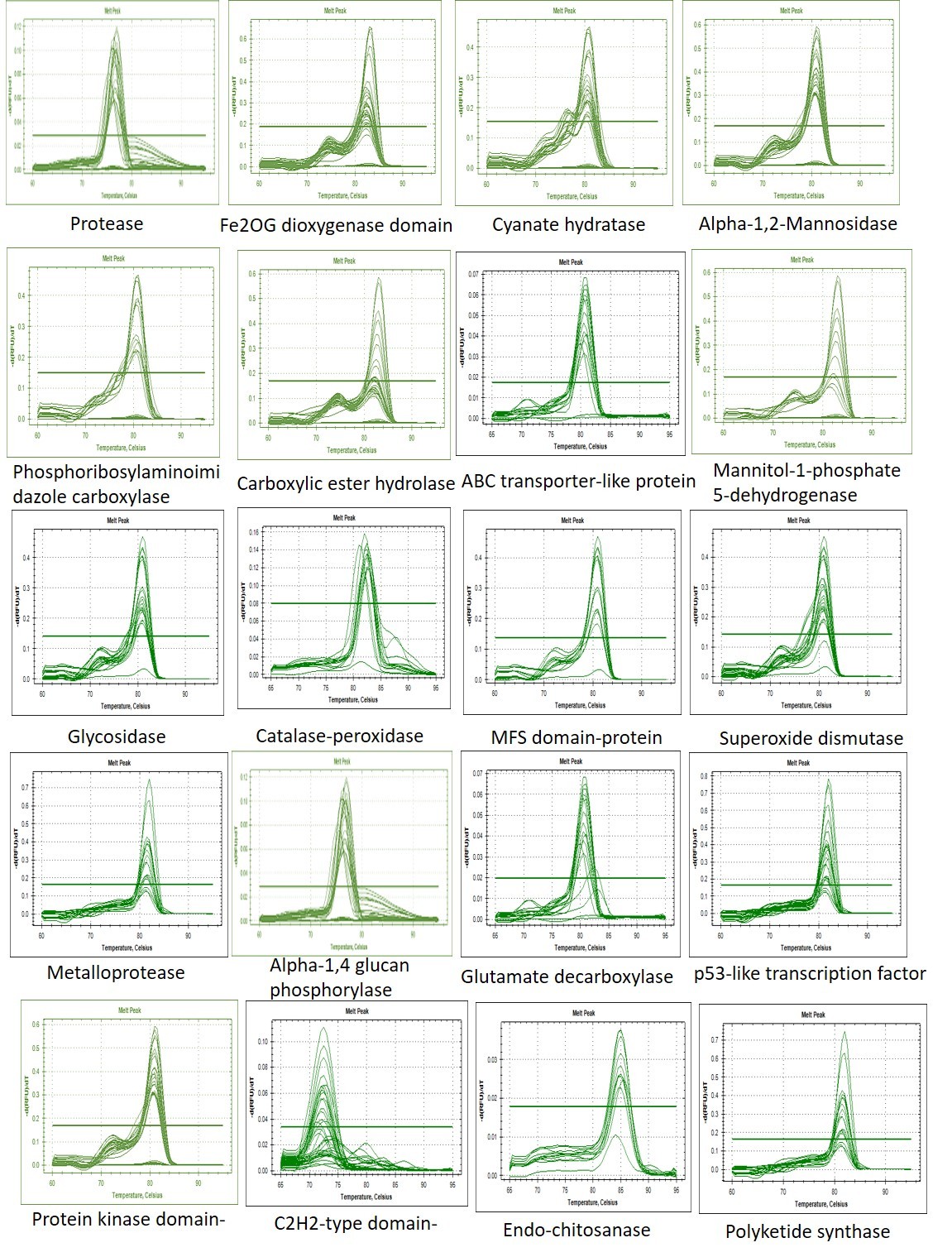

Supplement: Supplementary Figure 10 — Melting curves of different genes used for the real time PCR assays. [file Image_10.jpeg]
