# Supplementary material for: Transcriptome Profiling Provides Insights Into Potential Antagonistic Mechanisms Involved in Chaetomium globosum Against Bipolaris sorokiniana
Source: Front Microbiol. 2020 Dec 7;11:578115. doi: 10.3389/fmicb.2020.578115 (PMC7750538; doi:10.3389/fmicb.2020.578115)
Supplement: Supplementary Table 1 — RNA Quality and Concentration is estimated using Qubit 4.0 Fluorometer. [file Table_1.DOCX]

**Supplementary Table S1** RNA Quality and Concentration is estimated using Qubit 4.0 Fluorometer

| **SL NO** | **Sample ID** | **Sample Source** | **Conc.(ng/uL)** | **Total amount**  **of RNA available** | **RIN value** |
| --- | --- | --- | --- | --- | --- |
| 1 | Cg2_R1 | Mycelia | 1955 | 48875 | 7.60 |
| 2 | Cg2_R2 | Mycelia | 1010 | 25250 | 7.70 |
| 3 | Cg2 *BS112_R1 | Mycelia | 465 | 11625 | 7.32 |
| 4 | Cg2 *BS112_R2 | Mycelia | 500 | 12500 | 7.65 |
