# Supplementary material for: Transcriptome Profiling Provides Insights Into Potential Antagonistic Mechanisms Involved in Chaetomium globosum Against Bipolaris sorokiniana
Source: Front Microbiol. 2020 Dec 7;11:578115. doi: 10.3389/fmicb.2020.578115 (PMC7750538; doi:10.3389/fmicb.2020.578115)
Supplement: Supplementary Table 2 — List of validated primers used for Real time PCR. [file Table_2.DOCX]

**SupplementaryTable S2** The list of primers used for Real time PCR

| **SL no** | **Putative gene name** | **Primer name** | **Primer sequence (5’-3’)** | | **Primer length** | **Melting temperature (T_m_) °C** | **Product Size** |
| --- | --- | --- | --- | --- | --- | --- | --- |
| 1 | Protease | TRINITY_DN7674_c8_g3_i2 | F | CGCGAGCGGTACATTCATTG | 20 | 60.04 | 150 |
|  |  |  | R | CTGCATCTTGAGAGCGCAAC | 20 | 59.9 |  |
| 2 | Fe2OG dioxygenase domain-containing protein | TRINITY_DN7464_c5_g1_i4 | F | AGTTGCGAGTCGGTTGATGT | 20 | 59.97 | 189 |
|  |  |  | R | ATGTCCAAGCGGCTATCTCG | 20 | 59.97 |  |
| 3 | Cyanate hydratase (Cyanase) | TRINITY_DN7750_c11_g3_i1 | F | TTTGGGTAGGTTCGGTGACG | 20 | 59.97 | 198 |
|  |  |  | R | CGGGAGCAGTCTGGAACTTT | 20 | 59.96 |  |
| 4 | Alpha-1,2-Mannosidase | TRINITY_DN7658_c6_g5_i2 | F | ATTTCGAAGGCCCTGGTCTG | 20 | 60.04 | 228 |
|  |  |  | R | AACACTTCTCGTCACCCGTC | 20 | 59.97 |  |
| 5 | Phosphoribosylaminoimidazole carboxylase | TRINITY_DN7744_c3_g7_i2 | F | TCCTCAGTCCGGATCACCAT | 20 | 60.03 | 140 |
|  |  |  | R | GACGGGAGAGGCAACTTCAA | 20 | 59.97 |  |
| 6 | Carboxylic ester hydrolase | TRINITY_DN7946_c4_g3_i1 | F | TCGTACCGGGACACAACAAG | 20 | 59.97 | 156 |
|  |  |  | R | ACAACATCAAGAGCGGCAGA | 20 | 59.96 |  |
| 7 | ABC transporter-like protein | TRINITY_DN8092_c3_g3_i5 | F | TGTACTTTGGGGATGTCGGC | 20 | 60.04 | 133 |
|  |  |  | R | TTATTCACACCCTCCGCCAC | 20 | 60.04 |  |
| 8 | Mannitol-1-phosphate 5-dehydrogenase (M1PDH) | TRINITY_DN7971_c2_g2_i1 | F | TGGTACAAAGGAATGCCCCC | 20 | 59.96 | 197 |
|  |  |  | R | CGAAGATGACCTCGTACCCG | 20 | 59.97 |  |
| 9 | Glycosidase | TRINITY_DN7787_c5_g3_i1 | F | GGCATCGGTGAAGTCGGTAA | 20 | 60.11 | 202 |
|  |  |  | R | CATCAAGTGGACTCCCGACC | 20 | 60.11 |  |
| 10 | Catalase-peroxidase (CP)/ Peroxidase/catalase) | TRINITY_DN7146_c0_g3_i2 | F | CGATACGACGATGCTGGACA | 20 | 59.97 | 142 |
|  |  |  | R | TTTCCCAGTTGAGATCGGGC | 20 | 60.04 |  |
| 11 | MFS domain-containing protein | TRINITY_DN7412_c1_g1_i6 | F | AACTCGGGGTCAACTTTCCC | 20 | 59.89 | 163 |
|  |  |  | R | AATTGTCGGCAAGGTACGGT | 20 | 59.96 |  |
| 12 | Superoxide dismutase | TRINITY_DN7807_c0_g1_i1 | F | GCCCATTTTGGCGAGTGTTT | 20 | 59.97 | 150 |
|  |  |  | R | GACAAGAGACAAGCCCGGAA | 20 | 59.97 |  |
| 13 | Metalloprotease | TRINITY_DN7747_c4_g1_i2 | F | CCCGATCTTGTGAGCGCTAT | 20 | 59.97 | 174 |
|  |  |  | R | GTGTGAGAGTCACCGAAGCA | 20 | 59.97 |  |
| 14 | Glutamate decarboxylase | TRINITY_DN7551_c2_g4_i5 | F | CGGTCACCTTGTTCACCTCA | 20 | 59.89 | 196 |
|  |  |  | R | ACCGTTGCTAGAGCTTTGCT | 20 | 59.96 |  |
| 15 | Alpha-1,4 glucan phosphorylase | TRINITY_DN7581_c4_g1_i2 | F | GATCCGTCACGTTCCCACTT | 20 | 60.04 | 205 |
|  |  |  | R | CGTAGGAGTCCCAATCGTCG | 20 | 59.97 |  |
| 16 | P53-like transcription factor (Fragment) | TRINITY_DN7704_c1_g5_i10 | F | GACCGCCAAACCTGAGAAGA | 20 | 59.97 | 125 |
|  |  |  | R | CGAAGCCCTGGTCGTATCTC | 20 | 59.97 |  |
| 17 | Protein kinase domain-containing protein | TRINITY_DN7566_c2_g2_i12 | F | GTTCTGGGTCGTTCGGTGAT | 20 | 60.04 | 192 |
|  |  |  | R | TGGCGTTGTAGTCACACTCC | 20 | 59.97 |  |
| 18 | C2H2-type domain-containing protein | TRINITY_DN7776_c2_g8_i3 | F | GTAATAGTCCCAGGCGAGGC | 20 | 59.97 | 169 |
|  |  |  | R | TTCACACCAACGCCGTGATA | 20 | 59.97 |  |
| 19 | Endochitinase | TRINITY_DN7969_c3_g1_i1 | F | CTTAGCCTCTGAGACACCCG | 20 | 59.54 | 148 |
|  |  |  | R | GGTCTCACATGAATCGGCGT | 20 | 60.46 |  |
| 20 | Polyketide synthase | TRINITY_DN7358_c2_g1_i2 | F | CGCATCGGCAACGAATGAAT | 20 | 59.97 | 260 |
|  |  |  | R | ACGGCGTTTTTGATCTCCCT | 20 | 59.96 |  |
| 21 |  |  |  |  |  |  |  |
