# Supplementary material for: Transcriptome Profiling Provides Insights Into Potential Antagonistic Mechanisms Involved in Chaetomium globosum Against Bipolaris sorokiniana
Source: Front Microbiol. 2020 Dec 7;11:578115. doi: 10.3389/fmicb.2020.578115 (PMC7750538; doi:10.3389/fmicb.2020.578115)
Supplement: Supplementary Table 3 — Quality check statistics of transcriptomic data by FastQC. [file Table_3.DOCX]

**SupplementaryTable 3** Quality check statistics of transcriptomic data by FastQC

| **Samples** | **Raw reads** | **Clean reads** | **Q30(%)** | **Mapped reads** | **GC (%)** |
| --- | --- | --- | --- | --- | --- |
| **Cg2control_R1** | 23930890 | 23930890 | 94.23 | 23289542.15 | 58 |
| **Cg2control_R2** | 23842559 | 23842559 | 94.13 | 23842559 | 58 |
| **Cg2_BS112_R1** | 22377747 | 22377747 | 94.63 | 21921240.96 | 56.5 |
| **Cg2_BS112_R2** | 22070402 | 22070402 | 94.49 | 21611337.64 | 56.5 |
