# Supplementary material for: Transcriptome Profiling Provides Insights Into Potential Antagonistic Mechanisms Involved in Chaetomium globosum Against Bipolaris sorokiniana
Source: Front Microbiol. 2020 Dec 7;11:578115. doi: 10.3389/fmicb.2020.578115 (PMC7750538; doi:10.3389/fmicb.2020.578115)
Supplement: Supplementary Table 4 — Assembly statistics of transcriptome generated by Trinity (version 2.4.0) assembler. [file Table_4.DOCX]

**Supplementary Table 4** Assembly statistics of transcriptome generated by Trinity (version 2.4.0) assembler

| **Assembly details** | **Cg2** |
| --- | --- |
| Total transcripts (before clustered) | 55173 |
| Total transcripts (After clustered) | **45582** |
| Total assembled length (bp) | 105883810 |
| Average length | 2322.93 |
| GC% | 56.56 |
| Minimum transcript length (bp) | 201 |
| Largest transcripts length (bp) | 16285 |
| Contig >=1000 bp | 29309 |
| Contig >=5000 bp | 5022 |
| Contig >=10,000 bp | 453 |
| N50 length | **3805** |
| **Unigenes** |  |
| Total Number | **27957** |
| Total assembled (bp) | 51862109 |
| Average length | 1855.07 |
| GC% | 56.66 |
| N50 length | 3271 |
