# Supplementary material for: Transcriptome Profiling Provides Insights Into Potential Antagonistic Mechanisms Involved in Chaetomium globosum Against Bipolaris sorokiniana
Source: Front Microbiol. 2020 Dec 7;11:578115. doi: 10.3389/fmicb.2020.578115 (PMC7750538; doi:10.3389/fmicb.2020.578115)
Supplement: Supplementary Table 5 — List of Species distribution with most homologs to Chaetomium globosum. [file Table_5.DOCX]

**Supplementary Table S5** List of Species distribution with most homologues to *Chaetomium globosum*

| **Sl.No** | **Organism** | **Number of genes showed similarity** |
| --- | --- | --- |
|  | *Chaetomium globosum* (strain ATCC 6205) | 8434 |
|  | *Myceliophthora/ Sporotrichum thermophila* (strain ATCC 42464) | 3825 |
|  | *Thielavia terrestris* (*Acremonium alabamense*) | 1460 |
|  | *Madurella mycetomatis* | 1107 |
|  | *Metschnikowia bicuspidata* var. *bicuspidata* NRRL YB-4993 | 364 |
|  | *Podospora anserina* (strain S / FGSC 10383) (*Pleurage anserina*) | 201 |
|  | *Coniochaeta ligniaria* NRRL 30616 | 124 |
|  | *Chaetomium thermophilum* | 103 |
|  | *Chaetomium thermophilum* (strain DSM 1495 / IMI 039719) | 101 |
|  | *Magnaporthiopsis poae* (ATCC 64411) (*Magnaporthe poae*) | 51 |
|  | *Scedosporium apiospermum* | 48 |
|  | *Neurospora crassa* | 47 |
|  | *Sordaria macrospora* (ATCC MYA-333 / DSM 997 / K-hell) | 46 |
|  | *Gaeumannomyces graminis var. tritici* (strain R3-111a-1) | 45 |
|  | *Pestalotiopsis fici* (strain W106-1 / CGMCC3.15140) | 43 |
|  | *Neurospora crassa* (strain ATCC 24698/ FGSC 987) | 42 |
|  | *Togninia minima* (*Phaeoacremonium aleophilum*) | 40 |
|  | *Nectria haematococca* (*Fusarium solani subsp. pisi*) | 36 |
|  | *Lomentospora prolificans* | 34 |
|  | *Stagonospora* sp. SRC1lsM3a | 33 |
|  | *Phialocephala subalpina* | 33 |
|  | *Neonectria ditissima* | 32 |
|  | *Trichoderma harzianum* (*Hypocrealixii*) | 31 |
|  | *Stachybotrys chartarum* (strain CBS 109288) (*Stilbosporachartarum*) | 29 |
|  | *Valsa mali* | 28 |
|  | fungal sp. No.14919 | 27 |
|  | *Tolypocladium ophioglossoides* CBS 100239 | 26 |
|  | *Neurospora tetrasperma* (strain FGSC 2508/ ATCC MYA-4615 ) | 25 |
|  | *Pochonia chlamydosporia* 170 | 24 |
|  | *Gibberella nygamai* (*Fusarium nygamai*) | 24 |
|  | *Colletotrichum incanum* | 23 |
|  | *Hypocrea virens* (strain Gv29-8 ) (*Trichoderma virens*) | 22 |
|  | *Colletotrichum higginsianum* (strain IMI 349063) | 22 |
|  | *Tolypocladium paradoxum* | 21 |
|  | *Oidiodendron maius Zn* | 21 |
|  | *Endocarpon pusillum* (strain Z07020) (Lichen-forming fungus) | 21 |
|  | *Sporothrix insectorum* RCEF 264 | 20 |
|  | *Pyrenochaeta* sp. DS3sAY3a | 20 |
|  | *Phaeosphaeria nodorum* (strain SN15) (*Parastagonospora nodorum*) | 20 |
|  | *Hypoxylon* sp. EC38 | 19 |
