# Supplementary material for: Transcriptome Profiling Provides Insights Into Potential Antagonistic Mechanisms Involved in Chaetomium globosum Against Bipolaris sorokiniana
Source: Front Microbiol. 2020 Dec 7;11:578115. doi: 10.3389/fmicb.2020.578115 (PMC7750538; doi:10.3389/fmicb.2020.578115)
Supplement: Supplementary Table 7 — List of commonly expressed genes in Cg2-BS112 interaction. [file Table_7.DOCX]

**Supplementary material Table S7** The list of commonly expressed genes in Cg2-BS112 interaction

| **No.** | **Gene ID** | **Name** | **Log fold** | **Gene ontology** | **Function** |
| --- | --- | --- | --- | --- | --- |
|  | XP_003659416.1 | Glycosyltransferase family 39 protein | 16.65 | Integral component of membrane [GO:0016021]; mannosyltransferase activity [GO:0000030]; protein O-linked glycosylation [GO:0006493] | Metabolic pathways; Mannose type O-glycan biosynthesis; Other types of O-glycan biosynthesis; |
|  | AAY86760.1 | 60S ribosomal protein L7 | 16.41 | cytosolic large ribosomal subunit [GO:0022625]; structural constituent of ribosome [GO:0003735]; maturation of LSU-rRNA from tricistronic rRNA transcript |  |
|  | XP_018152220.1 | Histone H3 | 16.38 | nucleosome [GO:0000786]; nucleus [GO:0005634]; DNA binding [GO:0003677]; protein heterodimerization activity [GO:0046982] |  |
|  | XP_003664270.1 | Cytoplasmic tRNA 2-thiolation protein 1 (EC 2.7.7.-) \ | 16.30 | cytosol [GO:0005829]; nucleotidyltransferase activity [GO:0016779]; tRNA binding [GO:0000049]; protein urmylation [GO:0032447] |  |
|  | XP_001223273.1 | Mannitol-1-phosphate 5-dehydrogenase (EC 1.1.1.17) | 16.16 | coenzyme binding [GO:0050662]; mannitol-1-phosphate 5-dehydrogenase activity [GO:0008926]; mannitol metabolic process [GO:0019594] | Metabolic pathways; Fructose and mannose metabolism; |
|  | XP_003660446.1 | Uncharacterized protein | 15.54 | mitochondrion [GO:0005739]; ATP binding [GO:0005524]; ATPase activity [GO:0016887]; unfolded protein binding [GO:0051082]; protein folding [GO:0006457] |  |
|  | XP_003665138.1 | Glycosyltransferase family 48 protein | 15.43 | 1,3-beta-D-glucan synthase complex [GO:0000148]; integral component of membrane [GO:0016021]; 1,3-beta-D-glucan synthase activity [GO:0003843]; (1->3)-beta-D-glucan biosynthetic process [GO:0006075] | Metabolic pathways; Starch and sucrose metabolism; MAPK signaling pathway - yeast; |
|  | XP_001228473.1 | ADP,ATP carrier protein | 14.98 | integral component of membrane [GO:0016021]; mitochondrial inner membrane [GO:0005743]; ATP:ADP antiporter activity [GO:0005471]; mitochondrial ADP transmembrane transport [GO:0140021] | Calcium signaling pathway; cGMP-PKG signaling pathway; |
|  | XP_001220815.1 | Ribosome biogenesis protein ERB1 (Eukaryotic ribosome biogenesis protein 1) | 14.96 | nucleolus [GO:0005730]; nucleoplasm [GO:0005654]; preribosome, large subunit precursor [GO:0030687]; ribonucleoprotein complex binding [GO:0043021]; maturation of 5.8S rRNA [GO:0000466]; LSU-rRNA from tricistronic rRNA transcript [GO:0000463] |  |
|  | XP_003666573.1 | KOW domain-containing protein | 14.92 | large ribosomal subunit [GO:0015934]; structural constituent of ribosome [GO:0003735]; translation [GO:0006412] |  |
|  | XP_003653419.1 | UBA_e1_C domain-containing protein | 14.82 | cytoplasm [GO:0005737]; nucleus [GO:0005634]; ATP binding [GO:0005524]; magnesium ion binding [GO:0000287]; ubiquitin activating enzyme activity [GO:0004839] |  |
|  | XP_001219817.1 | Chitin synthase (EC 2.4.1.16) | 14.73 | integral component of membrane [GO:0016021]; chitin synthase activity [GO:0004100]; chitin biosynthetic process [GO:0006031] | Metabolic pathways; Amino sugar and nucleotide sugar metabolism; |
|  | XP_001222835.1 | GH16 domain-containing protein | 14.49 | integral component of membrane [GO:0016021]; hydrolase activity, hydrolyzing O-glycosyl compounds [GO:0004553]; carbohydrate metabolic process [GO:0005975] |  |
|  | XP_003649181.1 | Protein YOP1 | 14.32 | cell division site [GO:0032153]; cortical endoplasmic reticulum [GO:0032541]; integral component of membrane [GO:0016021]; nuclear envelope [GO:0005635]; endoplasmic reticulum inheritance [GO:0048309]; ER-dependent peroxisome organization [GO:0032581] |  |
|  | XP_003665954.1 | Carrier domain-containing protein | 14.17 | fatty acid synthase activity [GO:0004312]; holo-[acyl-carrier-protein] synthase activity [GO:0008897]; magnesium ion binding [GO:0000287]; long-chain fatty acid biosynthetic process [GO:0042759] | Metabolic pathways; Fatty acid metabolism; Fatty acid biosynthesis; |
|  | XP_003650886.1 | E3 ubiquitin-protein ligase (EC 2.3.2.26) | 14.13 | cellular bud tip [GO:0005934]; endosome membrane [GO:0010008]; Golgi apparatus [GO:0005794]; nucleus [GO:0005634]; cellular response to UV [GO:0034644]; mitochondrion organization [GO:0007005]; poly(A)+ mRNA export from nucleus [GO:0016973]; positive regulation of fatty acid biosynthetic process [GO:0045723] | Ubiquitin mediated proteolysis; MAPK signalling pathway - yeast; Endocytosis; |
|  | XP_003665506.1 | Coatomer subunit alpha | 14.02 | COPI vesicle coat [GO:0030126]; Golgi membrane [GO:0000139]; structural molecule activity [GO:0005198]; intracellular protein transport [GO:0006886] |  |
|  | KLO89647.1 | Putative EB1-like protein | 13.97 | microtubule [GO:0005874]; microtubule binding [GO:0008017] |  |
|  | AHL18116.1 | DNA-directed RNA polymerase subunit 2 | 13.87 | RNA polymerase II, core complex [GO:0005665]; DNA binding [GO:0003677];RNA polymerase II activity [GO:0001055] |  |
|  | XP_003666687.1 | V-type proton ATPase subunit | 13.75 | fungal-type vacuole membrane [GO:0000329]; proton-transporting V-type ATPase, V0 domain [GO:0033179]; proton-transporting ATPase activity, rotational mechanism [GO:0046961]; vacuolar transport [GO:0007034] |  |
