# Supplementary material for: Transcriptome Profiling Provides Insights Into Potential Antagonistic Mechanisms Involved in Chaetomium globosum Against Bipolaris sorokiniana
Source: Front Microbiol. 2020 Dec 7;11:578115. doi: 10.3389/fmicb.2020.578115 (PMC7750538; doi:10.3389/fmicb.2020.578115)
Supplement: Supplementary Table 8 — Gene ontology of differentially expressed genes, categorized into three sub categories, i.e., Biological process, molecular function, and cellular component. [file Table_8.DOCX]

**Supplementary Table S8** Gene ontology of differentially expressed genes, categorized into three sub categories i.e Biological process, molecular function and cellular component

| **GO-IDs** | **GO-term** | **Number of sequences** | |
| --- | --- | --- | --- |
|  |  | **Cg2 control** | **Cg2*BS112** |
| **Biological process** | | | |
| GO:0051179 | Localization | 410 | 436 |
| GO:0065007 | Biological regulation | 314 | 358 |
| GO:0008152 | Metabolic process | 1420 | 1422 |
| GO:0009987 | Cellular process | 1465 | 1514 |
| GO:0050896 | Response to stimulus | 232 | 245 |
| GO:0050789 | Regulation of biological process | 274 | 312 |
| GO:0048519 | Negative regulation of biological process | 66 | 75 |
| GO:0071840 | Cellular component organization / biogenesis | 324 | 348 |
| GO:0023052 | Signalling | 91 | 98 |
| GO:0048518 | Positive regulation of biological process | 59 | 71 |
| GO:0051704 | Multi-organism process | 14 | 14 |
| GO:0002376 | Immune system process | 01 | 01 |
| GO:0022414 | Reproductive process | 26 | 29 |
| GO:0000003 | Reproduction | 26 | 29 |
| GO:0032502 | Developmental process | 18 | 21 |
| GO:0015976 | Carbon utilization | 01 | 02 |
| GO:0019740 | Nitrogen utilization | 03 | 03 |
| **MolecularFunction** | | | |
| GO:0003824 | Catalytic activity | 2056 | 2033 |
| GO:0005215 | Transporter activity | 221 | 213 |
| GO:0005488 | Binding | 1920 | 1910 |
| GO:0060089 | Molecular transducer activity | 08 | 11 |
| GO:0016209 | Antioxidant activity | 15 | 17 |
| GO:0045182 | Translation regulator activity | 02 | 01 |
| GO:0098772 | Molecular function regulator | 56 | 58 |
| GO:0005198 | Structural molecule activity | 73 | 80 |
| GO:0045735 | Nutrient reservoir activity | 01 | 03 |
| GO:0140104 | Molecular carrier activity | 04 | 05 |
| GO:0140110 | Transcription regulator activity | 181 | 193 |
| GO:0038024 | Cargo receptor activity | 02 | 02 |
| **Cellular Component** | | | |
| GO:0005623 | Cell | 1253 | 1313 |
| GO:0044464 | Cell part | 1213 | 1266 |
| GO:0044422 | Organelle part | 534 | 522 |
| GO:0043226 | Organelle | 994 | 1027 |
| GO:0044425 | Membrane part | 1338 | 1248 |
| GO:0016020 | Membrane | 1415 | 1314 |
| GO:0032991 | Protein-containing complex | 520 | 548 |
| GO:0099080 | Supramolecular complex | 17 | 19 |
| GO:0031974 | Membrane-enclosed lumen | 181 | 194 |
| GO:0005576 | Extracellular region | 26 | 19 |
| GO:0009295 | Nucleoid | 4 | 4 |
