# Supplementary material for: Transcriptome Profiling Provides Insights Into Potential Antagonistic Mechanisms Involved in Chaetomium globosum Against Bipolaris sorokiniana
Source: Front Microbiol. 2020 Dec 7;11:578115. doi: 10.3389/fmicb.2020.578115 (PMC7750538; doi:10.3389/fmicb.2020.578115)
Supplement: Supplementary Table 10 — KEGG Pathway enrichment analysis of differentially expressed genes. [file Table_10.DOCX]

**Supplementary Table S10** KEGG Pathway enrichment analysis of differentially expressed genes

| **KEGG pathways** | **p-value** | **Number of genes** | |
| --- | --- | --- | --- |
|  |  | **Cg2 control** | **Cg2*BS112** |
| Metabolic pathways | 2.2E-16 | 744 | 1062 |
| Biosynthesis of secondary metabolites | 2.2E-16 | 261 | 382 |
| Biosynthesis of antibiotics | 2.2E-16 | 220 | 269 |
| Microbial metabolism in diverse environments | 4.965E-15 | 174 | 223 |
| Cell cycle | 1.798E-11 | 81 | 219 |
| Ribosome | 1.065E-08 | 220 | 145 |
| Biosynthesis of amino acids | 4.788E-12 | 80 | 143 |
| Carbon metabolism | 3.463E-16 | 94 | 126 |
| MAPK signaling pathway | 1.541E-12 | 73 | 109 |
| RNA transport | 2.615E-07 | 71 | 102 |
| Protein processing in endoplasmic reticulum | 2.203E-12 | 69 | 99 |
| Starch and sucrose metabolism | 3.344E-010 | 32 | 66 |
